# Nuclear connectin novex-3 promotes proliferation of hypoxic foetal cardiomyocytes

**DOI:** 10.1038/s41598-018-30886-9

**Published:** 2018-08-17

**Authors:** Ken Hashimoto, Aya Kodama, Miki Sugino, Tomoko Yobimoto, Takeshi Honda, Akira Hanashima, Yoshihiro Ujihara, Satoshi Mohri

**Affiliations:** 1 First Department of Physiology, Kawasaki Medical School, Kurashiki, Japan; 2 Department of Cardiovascular Surgery, Kawasaki Medical School, Kurashiki, Japan

## Abstract

Loss of cardiomyocyte proliferative capacity after birth is a major obstacle for therapeutic heart regeneration in adult mammals. We and others have recently shown the importance of hypoxic in utero environments for active foetal cardiomyocyte proliferation. Here, we report the unexpected expression of novex-3, the short splice variant of the giant sarcomeric protein connectin (titin), in the cardiomyocyte nucleus specifically during the hypoxic foetal stage in mice. This nuclear localisation appeared to be regulated by the N-terminal region of novex-3, which contains the nuclear localisation signal. Importantly, the nuclear expression of novex-3 in hypoxic foetal cardiomyocytes was repressed at the postnatal stage following the onset of breathing and the resulting elevation of oxygen tension, whereas the sarcomeric expression remained unchanged. Novex-3 knockdown in foetal cardiomyocytes repressed cell cycle-promoting genes and proliferation, whereas novex-3 overexpression enhanced proliferation. Mechanical analysis by atomic force microscopy and microneedle-based tensile tests demonstrated that novex-3 expression in hypoxic foetal cardiomyocytes contributes to the elasticity/compliance of the nucleus at interphase and facilitates proliferation, by promoting phosphorylation-induced disassembly of multimer structures of nuclear lamins. We propose that novex-3 has a previously unrecognised role in promoting cardiomyocyte proliferation specifically at the hypoxic foetal stage.

## Introduction

While foetal cardiomyocytes (fCMs) in mammals actively proliferate in utero to form the primitive heart, they stop dividing soon after birth. For this reason, understanding the molecular mechanisms of active fCM proliferation is fundamental for therapeutic regeneration in adult hearts. Recently, we and others have shown that activation of fCM proliferation requires a low oxygen (O_2_) condition in foetal hearts, prior to the onset of breathing at birth^1,2^. However, the underlying molecular mechanism of this activation is unknown. One study has shown that proliferation of mouse fCMs during the mid-embryonic stage (embryonic day, E12.5–E14.5) was maintained by hypoxia inducible factor-1α (Hif-1α)^3^. We have recently identified family with sequence similarity 64, member A (Fam64a; also known as Pimreg) as an essential molecule for hypoxic fCM proliferation^2^. However, the regulatory networks occurring among these and other unidentified molecules under hypoxic foetal conditions remain poorly understood.

Connectin (also known as titin; Ttn) is the largest protein discovered to date (3~4 MDa) and spans from the Z-disk, I-band and A-band to the M-band region of the sarcomere of cardiac and skeletal muscle^4,5^. It has 4 major isoforms, N2A, N2B, N2BA and foetal cardiac connectin, as well as numerous isoforms, including shorter fragments that are produced through complex alternative splicing pathways. Generally, connectin functions as an elastic molecular spring, and in cardiac muscle, it defines myocardial passive stiffness in diastole. However, connectin has recently been recognised to have additional functions, including acting as a structural and signalling molecule^6^.

Our recent work on connectins has focused on their nuclear localisation. In *C. elegans* embryos, two connectin homologues, TTN-1/Ce-titin (2.2 MDa) and kettin (500–550 KDa), were detected at the interphase nuclear envelope^7^, and kettin was also localised to mitotic spindles during mitosis. A similar nuclear localisation of kettin was observed in very early embryos^8,9^. In *Drosophila* embryos, a nuclear connectin was identified that differed from the major isoform found in the muscle sarcomere^10,11^. This protein was 1.9 MDa in size and showed significant homology to the N-terminal half of vertebrate connectin; it was named D-titin (also called Sls protein^12^ or I-connectin^13^). The nuclear and sarcomeric isoforms of D-titin are proposed to be distinct splice variants from the same gene, where the former lacks the N-terminal regions, called Z-repeats, which bind to α-actinin in the Z-disk. The nuclear isoform functions in the assembly of condensed chromosomes and provides elasticity to those structures during mitosis. D-titin has also been found in the nuclear envelope, mitotic spindles and chromosomes during mitosis in insect spermatocytes, where it was suggested to provide elasticity to the spindle matrix^14^. Interestingly, kettin is the N-terminal short variant of D-titin^12^, and it lacks Z-repeats^15^. Moreover, the epitope of α-KZ, the antibody used to detect nuclear D-titin, is located in the kettin region of the D-titin gene^11^. In mammals, several studies have shown that antibodies against multiple connectin epitopes label the nucleus in various non-muscle cell lines^7,10,16,17^. The molecular basis for the nuclear localisation of connectin was demonstrated by the discovery of a nuclear localisation signal (NLS) located at the N-terminus between the Z2 and Z repeats (200-PAKKTKT)^17^; this NLS is shared by all connectin isoforms.

Certain of these nuclear isoforms of connectins have been implicated as cell cycle promoters^11,17–19^. The N-terminal short fragment of connectin was expressed in the nucleus of human osteoblastic cells, and promoted proliferation by activating the Wnt/β-catenin pathway^17^. In *Drosophila*, mutations in D-titin cause chromosome under-condensation, chromosome breakage, loss of diploidy and premature sister chromatid separation in neuroblasts, as well as defects in muscle development^11^. However, these nuclear functions have all been reported to date in non-muscle cells.

In the present study, we investigated whether the nuclear connectin is functional in muscle cells and whether it is linked to fCM cell cycle regulation in perinatal hearts in mammals. We focused on the novex-3 isoform, which shares the N-terminal region with the full connectin molecule, including the NLS and Z-repeats, but has an alternative termination signal encoded by the large unique exon, making it a shorter, truncated ~650 kDa connectin isoform^20^. Novex-3 was first identified in 2001^20^, but only a few studies have yet investigated its functions. The first study showed that novex-3 integrates into the Z-disk lattice and interacts with obscurin, a connectin-related protein, which together provides elasticity to the sarcomere^20^. A further study demonstrated that the prominent expression of novex-3 homologue in early *Xenopus laevis* embryo later declined at the tadpole stages, and this decline was associated with the switch from a primary myogenesis to a secondary myogenesis^21^. In the present study, we found that a considerable amount of novex-3 is expressed in the CM nuclei in mice, specifically at the hypoxic foetal stage before birth. This nuclear expression provides elasticity/compliance to the interphase CM nuclei, and contributes to active fCM proliferation.

## Results

### Novex-3 is localised in the nucleus of mouse fCMs

**Figure 1:**
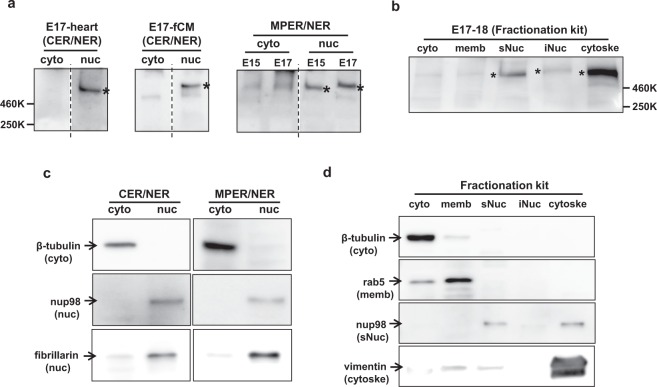
Novex-3 is localised in the nucleus of mouse fCMs. (**a**) A representative immunoblot for novex-3 protein (marked with *) from foetal mouse heart tissues (left and right) or cultured mouse fCMs (middle, E17). The subcellular fractionation of proteins was done by NE-PER system, which uses CER buffer for cytoplasmic extraction and NER buffer for nuclear extraction (left and middle). In some samples, CER buffer was replaced with MPER buffer to validate the result of different fractionation methods (right; see Methods). The broken line between the lanes separates the samples from different parts of the same blot. (**b**) Detailed fractionation analysis was performed to obtain five fractions, i.e. cytoplasmic, membrane, soluble nuclear (sNuc), insoluble chromatin-bound nuclear (iNuc), and cytoskeletal fractions, which were used to probe novex-3 protein (marked with *) by immunoblotting. (**c**) The two fractionation methods used in (**a**) were validated by immunoblotting a specific protein with known localisation: β-tubulin (cytoplasmic), nup98 (nuclear) and fibrillarin (nuclear). (**d**) The fractionation method used in (**b**) was validated by immunoblotting a specific protein with known localisation: β-tubulin (cytoplasmic), rab5 (membrane), nup98 (nuclear) and vimentin (cytoskeletal).

In the various tissues examined, the expression of novex-3 protein was restricted to striated muscle, as previously described (Supplementary Fig. S1). Western blot analysis of foetal mouse hearts (embryonic day, E15–E18) revealed novex-3 protein as a clear single band of ~650 kDa (Fig. 1a,b). Importantly, the full size novex-3 was highly enriched in the nuclear fraction but was not detected in the cytoplasmic fraction, both in the samples from foetal heart tissues (Fig. 1a, left) and in cultured fCMs (Fig. 1a, middle). The different fractionation methods gave similar results (Fig. 1a, right). Detailed fractionation analysis further revealed the presence of novex-3 in both the nuclear and cytoskeletal fractions (Fig. 1b). These data indicate that novex-3 protein is localised in CM nuclei in foetal hearts, in addition to its well-known sarcomeric localisation. In the CM nuclei, novex-3 was mostly found in the soluble fraction, which represents the nucleoplasm and nuclear envelope, but it was also found marginally in chromatin-bound insoluble fraction (Fig. 1b). The fractionation methods were verified by identification of specific marker proteins for each fraction (Fig. 1c,d).

### Novex-3 nuclear localisation appears to be regulated by the NLS in the N-terminal region

**Figure 2:**
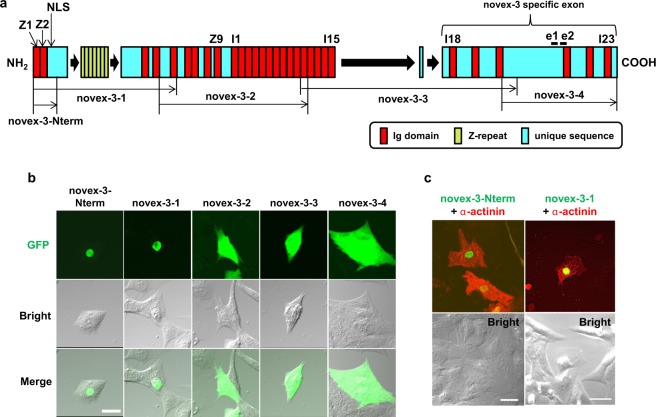
Novex-3 nuclear localisation appears to be regulated by the nuclear localisation signal (NLS) in the N-terminal region. (**a**) Primary structure of human novex-3 (assembled from refs^20,36^). Mouse novex-3 has a similar molecular structure (Supplementary Fig. S9). The epitope of the two different antibodies used in this study is marked with “e1” and “e2”, both of which is within the novex-3-specific exon (see Methods). The nuclear localisation signal (NLS) located at N-terminus between Z2 and Z repeats^17^ is shown. (**b**) Four fragments covering the full length novex-3 (novex-3-1 through novex-3–4) as well as novex-3-Nterm fragment containing connectin exons 1 to 6 (shown in (**a**)), were independently expressed as N-terminal EGFP-tagged proteins in cultured fCMs (E16–E18) by baculovirus-mediated protein expression system. Live cells were observed by fluorescence microscopy together with bright-field optics. The approximate protein size of these five fragments, excluding the tagged-EGFP (~27 kDa), was 146, 206, 170, 146 and 32.6 kDa for novex-3–1, novex-3-2, novex-3-3, novex-3–4 and novex-3Nterm, respectively. (**c**) The E16 fCMs doubly expressing novex-3-Nterm-GFP and sarcomeric α-actinin-mCherry (left), or novex-3-1-GFP and sarcomeric α-actinin-mCherry (right), were shown together with bright-field images. Sarcomeric α-actinin was used as a specific CM marker. Scale bars = 20 µm in (**b**) and 30 µm in (**c**).

We used our baculovirus-mediated protein expression system^2^ for the independent expression of four fragments covering the full length novex-3 (novex-3-1 through novex-3–4; Fig. 2a) as GFP fusion proteins in cultured fCMs. Figure 2b shows that novex-3-1, which contains the NLS, was localised exclusively in the nucleus, while the other three fragments exhibited diffuse cytoplasmic staining. The shorter fragment containing connectin exons 1 to 6 (novex-3-Nterm), which contains the NLS, also displayed nuclear localisation. The CMs were identified by their morphological appearance in bright-field images, their beating characteristics in live cell observations and the striated staining of sarcomeric α-actinin (as a CM marker), which was co-expressed as an mCherry-tagged protein (Fig. 2c). These data suggest that novex-3 nuclear localisation is regulated by the NLS in the N-terminal region.

### Nuclear expression of novex-3 in hypoxic fCMs is repressed after birth

**Figure 3:**
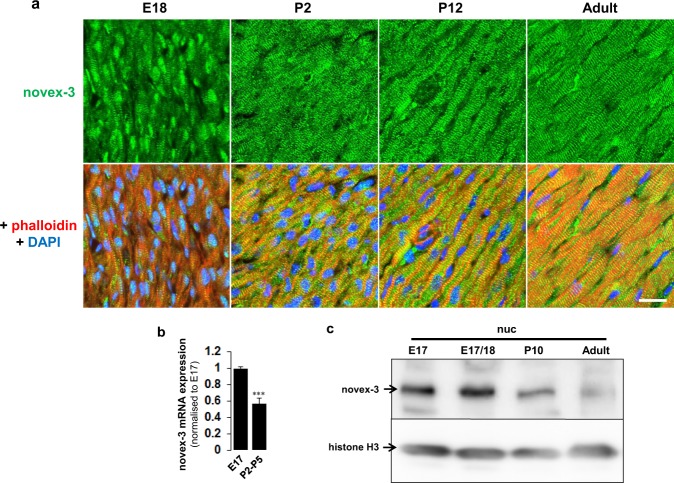
Nuclear expression of novex-3 in hypoxic fCMs is repressed after birth. (**a**) Immunofluorescence staining for novex-3 (green) in phalloidin (red) and DAPI (blue)-stained mouse heart sections at indicated stages. E; embryonic day, P; postnatal day. Scale bar = 20 µm. (**b**) qPCR analysis of novex-3 mRNA expression in mouse hearts at indicated stages. n = 4–6 independent experiments. ***P < 0.001 compared to E17. Error bar = SEM. (**c**) A representative immunoblot for novex-3 protein in nuclear fraction from mouse hearts at indicated stages. Histone H3 was included as a loading control.

We used immunofluorescence studies of heart tissues to confirm that novex-3 protein was amply expressed in the nucleus of E18 fCMs, as well as in the sarcomere (Fig. 3a). Staining of isolated fCM nuclei further confirmed the presence of novex-3 protein in the nuclei (Supplementary Fig. S2). However, this nuclear expression was almost completely repressed after birth, when O_2_ tension was elevated by the onset of breathing (Fig. 3a; P, postnatal day). By contrast, the sarcomeric expression remained unchanged (Fig. 3a and Supplementary Fig. S3). Biochemical analysis demonstrated a significant decline in the novex-3 mRNA level (Fig. 3b) and nuclear novex-3 protein expression (Fig. 3c) after birth.

### Nuclear expression of novex-3 in hypoxic fCMs is repressed by exposure to higher O_2_

**Figure 4:**
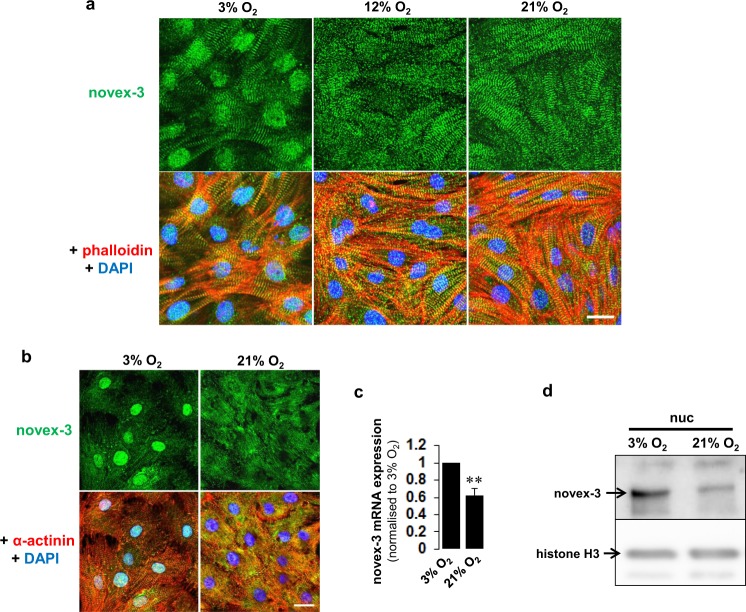
Nuclear expression of novex-3 in hypoxic fCMs is repressed by exposure to higher O_2_. (**a**) Immunofluorescence staining for novex-3 (green) observed in phalloidin (red) and DAPI (blue) in E17 fCMs cultured under different O_2_ conditions. Scale bar = 20 µm. (**b**) Immunofluorescence staining for novex-3 (green, another e2 antibody was used; see Methods) observed in sarcomeric α-actinin (red, as a CM marker) and DAPI (blue) in E17 fCMs cultured under different O_2_ conditions. Scale bar = 20 µm. (**c**) qPCR analysis of novex-3 mRNA expression in fCMs cultured under different O_2_ conditions. n = 3 independent experiments. **P < 0.01 compared to 3% O_2_. Error bar = SEM. (**d**) Immunoblot analysis for novex-3 protein in nuclear fraction from E17 fCMs cultured under different O_2_ conditions. Histone H3 was included as a loading control.

We next examined whether the reduction in nuclear novex-3 expression after birth is caused by increased O_2_ levels. We mimicked the truly hypoxic intrauterine O_2_ conditions (20–25 mmHg; 2.6–3.2% O_2_)^22^ in our fCM culture using our established cell isolation protocol performed under strict low O_2_ conditions^2^. Isolated fCMs were separately cultured under different O_2_ conditions for 48–96 h. As shown in Fig. 4a, nuclear expression of novex-3 was observed in fCMs under low O_2_ conditions (3% O_2_). By contrast, a considerable fraction of fCMs showed repression of this nuclear expression following exposure to higher O_2_ levels during culture (12–21% O_2_), whereas the sarcomeric expression remained unchanged. We confirmed this observation with another novex-3 antibody that detects nuclear signals more intensely (Fig. 4b). Biochemical analysis confirmed these results for both the mRNA and the protein levels (Fig. 4c,d). We confirmed that *in vitro* culture itself did not affect the expression pattern of novex-3 in fCMs (Supplementary Fig. S4).

### Nuclear novex-3 is a cell cycle promoter in hypoxic fCMs

**Figure 5:**
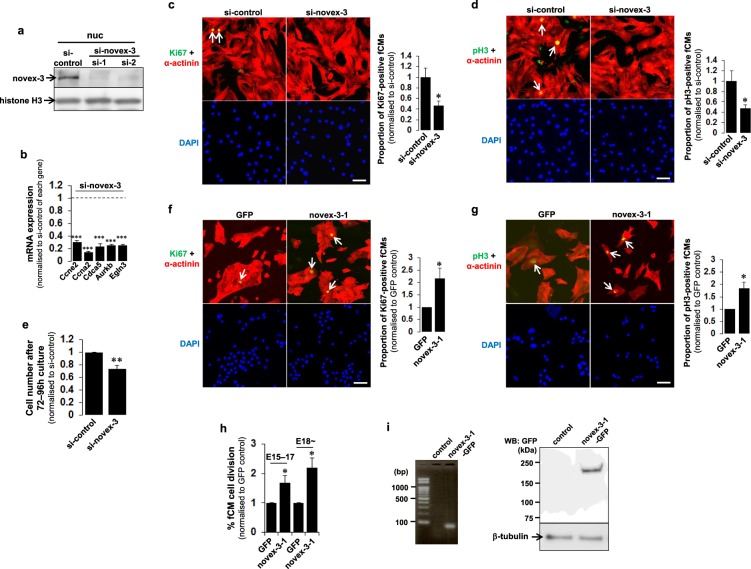
Nuclear novex-3 is a cell cycle promoter in hypoxic fCMs. (**a**) Novex-3 was silenced in E17 fCMs by siRNA, and the nuclear fraction was used to probe novex-3 protein by immunoblotting. Two siRNAs specific for novex-3, designated as si-1 and si-2, were used. Histone H3 was included as a loading control. (**b**) qPCR analysis of mRNA levels of the indicated genes in novex-3-silenced fCMs (E16–E17). Data are shown as normalised to the si-control level of each gene, set at 1. n = 3 independent experiments. ***P < 0.001 compared to the si-control level of each gene. (**c**,**d**) Immunofluorescence for Ki67 (**c**, green) and phospho-histone H3 (pH3) (**d**), green) observed in sarcomeric α-actinin (red, as a CM marker) and DAPI (blue) in novex-3-silenced fCMs (E17). Arrows denote Ki67-positive (**c**) and pH3-positive (**d**) fCMs. Proportions of Ki67-positive (**c**) and pH3-positive (**d**) fCMs were shown on the right. n = 4 independent experiments. *P < 0.05 compared to si-control. Scale bars = 50 µm. (**e**) Proliferative activity of novex-3-silenced E16–E17 fCMs evaluated by cell counting after 72–96 h culture. n = 3 independent experiments. **P < 0.01 compared to si-control. (**f**,**g**) Immunofluorescence for Ki67 (**f**), green) and phospho-histone H3 (pH3) (**g**, green) observed in sarcomeric α-actinin (red, as a CM marker) and DAPI (blue) in novex-3-1-overexpressing fCMs (E15–E17). Arrows denote Ki67-positive (**f**) and pH3-positive (**g**) fCMs. Proportions of Ki67-positive (**f**) and pH3-positive (**g**) fCMs are shown at right. n = 4 independent experiments. *P < 0.05 compared to control GFP-overexpressing fCMs. Scale bars = 50 µm. (**h**) Percentage of novex-3-1-overexpressing fCMs that completed cell division, as determined by time-lapse imaging. Analysis was performed separately in two developmental stages (E15–E17 and later than E18). n = 3 independent experiments. *P < 0.05 compared to control GFP-overexpressing fCMs. (**i**) Left, overexpression of novex-3-1-GFP mRNA in E17 fCMs was confirmed by probing 61 bp amplicon within GFP sequence by PCR. Right, overexpression of novex-3-1-GFP protein in E17 fCMs was confirmed by immunoblotting the whole protein extracts using the antibody against GFP. A clear single band was observed at the expected molecular mass (novex-3-1: ~175 kDa + GFP: ~27 kDa). β-tubulin was included as a loading control. Error bars = SEM.

The role of novex-3 in the proliferation of hypoxic fCMs was examined by siRNA-mediated knockdown, which resulted in almost complete depletion (>90%) of the novex-3 protein in the nuclear extracts (Fig. 5a). We confirmed that these two siRNAs produce no toxicity or apoptosis-inducing effects on fCMs (Supplementary Fig. S5). Therefore, subsequent experiments were performed using either of the two siRNAs. Novex-3 knockdown in hypoxic fCMs drastically suppressed the expression of major cell cycle-promoting genes, including *Ccne2*, *Ccna2*, *Cdca5* and *Aurkb*, to levels that were 10–30% those of the negative control siRNA (Fig. 5b). Knockdown of novex-3 significantly decreased the proportion of fCMs showing positivity for Ki67, a cell cycle marker (Fig. 5c), and phospho-histone H3 (pH3), a mitosis marker (Fig. 5d). These reductions in cell cycle activity resulted in a significant inhibition of fCM proliferation (Fig. 5e). Conversely, overexpression of the novex-3-1 fragment significantly increased the proportion of fCMs showing positivity for Ki67 (Fig. 5f) and pH3 (Fig. 5g). Time-lapse imaging analysis revealed that overexpression of this fragment enhanced fCM proliferation at both early (E15–E17) and later (E18~) embryonic stages (Fig. 5h). Overexpression of this fragment was confirmed at both the mRNA and protein levels (Fig. 5i). These data indicate that novex-3 serves as a cell cycle promoter in hypoxic fCMs.

### Nuclear function of novex-3 as a cell cycle promoter is ascribed to that in interphase, but not during mitosis

**Figure 6:**
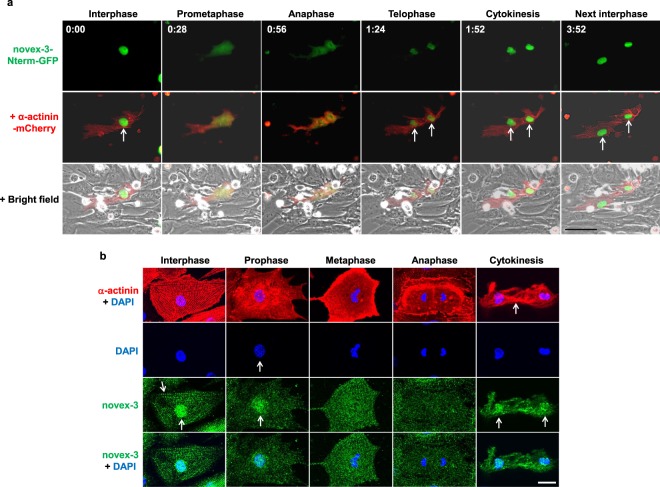
Nuclear function of novex-3 as a cell cycle promoter is ascribed to that in interphase, but not during mitosis. (**a**) A representative time-lapse recording of cell division dynamics of the E16 fCM doubly expressing novex-3-Nterm-GFP and sarcomeric α-actinin-mCherry (as a CM marker). In contrast to the clear nuclear expression observed during interphase (arrow), novex-3-Nterm expression was not localised to specific structures (spindles, spindle matrix, centrioles, or chromosomes) during mitosis; instead, it was diffusely spread throughout the cytoplasm following nuclear envelope breakdown at prometaphase. At telophase, when the nuclear envelope reassembled, the novex-3-Nterm re-accumulated in the newly formed daughter nuclei (arrows in telophase and cytokinesis), where it was maintained over the next interphase (arrows). The number on each panel indicates the time in (hours: minutes) elapsed from the time indicated in the first panel. Scale bar = 50 µm. (**b**) Cultured fCMs (E17–E18) were triple-stained with novex-3, sarcomeric α-actinin (as a CM marker), and DAPI. DAPI staining clearly defined each phase in mitosis, including prophase, metaphase, anaphase, and subsequent cytokinesis. In interphase fCMs, novex-3 was expressed both in nuclei and sarcomere (arrows). At this time point, sarcomere structure was intact, as indicated by α-actinin staining. In prophase, when chromosome condensation was visible as dot-like puncta by DAPI staining (arrow), novex-3 was still expressed in fCM nuclei (arrow), while sarcomere structure has begun to disassemble. After the nuclear envelope breakdown at prometaphase, novex-3 did not localise to the specific structures such as spindles, spindle matrix, centrioles, or chromosomes; instead it diffused throughout the cytoplasm. When the nuclear envelope reassembled during cytokinesis, novex-3 re-accumulated in the newly formed daughter nuclei (arrows). Scale bar = 20 µm.

The promotion of fCM proliferation by nuclear novex-3 was further examined in fCMs expressing both novex-3-Nterm fragment and sarcomeric α-actinin (as a CM marker) using time-lapse analysis, which enables detailed kinetic analysis of novex-3-Nterm distribution during cell division in living fCMs. As shown in Fig. 6a, in contrast to the clear nuclear expression observed during interphase, novex-3-Nterm expression was not localised to specific structures (spindles, spindle matrix, centrioles, or chromosomes) during mitosis; instead, it was diffusely spread throughout the cytoplasm following nuclear envelope breakdown at prometaphase. At telophase, when the nuclear envelope reassembled, the novex-3-Nterm re-accumulated in the newly formed daughter nuclei, where it was maintained over the next interphase (See also Supplementary Movies 1–3). An immunofluorescence study in fixed cells gave similar results (Fig. 6b), confirming that the full-size novex-3 behaves similarly to the novex-3-Nterm fragment in live cell analysis during fCM cell division. These results suggest that the nuclear function of novex-3 as a cell cycle promoter likely occurs during interphase, but not during mitosis.

### Novex-3 plays a role in providing the elasticity/compliance to the fCM nuclei by promoting phosphorylation-induced disassembly of lamin multimer structures

**Figure 7:**
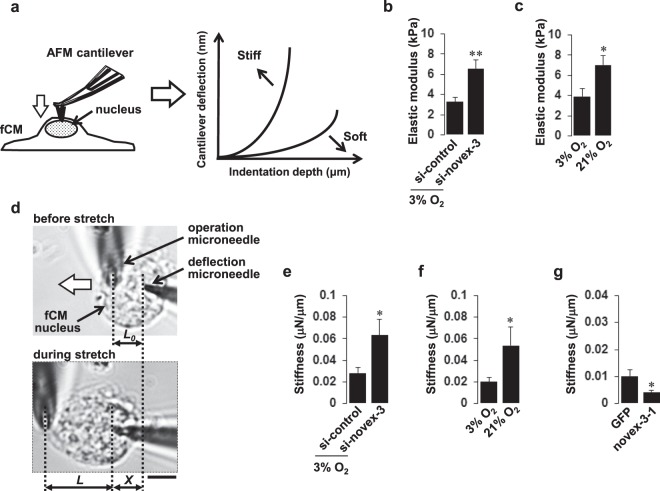
Novex-3 plays a role in providing the elasticity/compliance to the fCM nuclei. (**a**) Mechanical indentation measurements by atomic force microscopy (AFM). Intrusion of the AFM cantilever onto the surface of the fCM generated a force-indentation curve, which is the relationship between the cantilever deflection and its indentation depth. The elastic modulus (**E**) was calculated based on the Hertz model (see Methods). Measurements were performed on the central region of the nuclei, as monitored by phase contract optics as previously described^26–28^. (**b**) The elastic modulus of novex-3-silenced E16 fCMs evaluated on the central region of the nuclei. fCMs were cultured under 3% O_2_ condition. n = 6–8 fCMs. **P < 0.01 compared to si-control. Error bars = SEM. (**c**) The elastic modulus of E17 fCMs cultured under different O_2_ conditions evaluated on the central region of the nuclei. n = 6–7 fCMs. *P < 0.05 compared to 3% O_2_ condition. Error bars = SEM. (**d**) Microneedle-based tensile tests. One microneedle, designated as an operation microneedle, was rigid and was moved with a three-axis motorised micromanipulator to stretch the nucleus. The other microneedle, designated as a deflection microneedle, was flexible, to obtain the force applied to the nucleus by measuring its deflection. A single isolated fCM nucleus was captured by putting the microneedle tips into the nucleus. The nucleus was then lifted off the chamber bottom and stretched horizontally at a rate of 1 μm/s by moving the operation microneedle along the surface of the chamber bottom. The stiffness was calculated from the slope of the force-deformation curve. *L*, the distance between the tips of the two microneedles along the axis of stretch (*L*_0_, the initial distance before stretch). *X*, the deflection of the deflection microneedle. Scale bar = 5 µm. (**e**) Stiffness of novex-3-silenced fCM nuclei isolated from E17–E18 fCMs cultured under 3% O_2_ condition. n = 6–13 nuclei. *P < 0.05 compared to si-control. Error bars = SEM. (**f**) Stiffness of fCM nuclei isolated from E17–E18 fCMs cultured under different O_2_ conditions. n = 6–9 nuclei. *P < 0.05 compared to 3% O_2_ condition. Error bars = SEM. (**g**) Stiffness of novex-3–1-overexpressing fCM nuclei isolated from E17 fCMs cultured under 3% O_2_ condition. n = 7–9 nuclei. *P < 0.05 compared to control GFP-overexpressing fCM nuclei. Error bars = SEM.

Connectins generally function by providing elasticity to the sarcomere^4,20^, and a similar elastic function has been proposed in the nucleus in non-muscle cells^7,10,11,14^. We therefore investigated the involvement of novex-3 in regulating the elasticity of interphase fCM nuclei by mechanical indentation measurements with atomic force microscopy (AFM) (Fig. 7a). AFM is a valuable tool for quantifying the mechanical properties of live, intact cells with high resolution^23–25^, and it provides the elastic moduli (indicators of the stiffness) for various intracellular target regions, including the nucleus and cytoplasm^26–28^. Novex-3 knockdown significantly increased the elastic modulus of the interphase fCM nuclei (Fig. 7b), indicating that fCM nuclei became stiffer (i.e., had a reduced elasticity) when novex-3 expression was repressed. A reduced elasticity was also observed in the interphase nuclei following exposure to elevated O_2_ (Fig. 7c).

We excluded the possible contribution of non-nuclear components, such as cytosol and/or membranes, in our AFM measurements by performing experiments on a single isolated fCM nucleus and stretching it directly with a microneedle-based tensile test system to obtain the true stiffness of the nucleus (Fig. 7d). The results were similar to those obtained with whole fCM cells (Fig. 7e,f and Supplementary Fig. S6). Conversely, overexpression of the novex-3-1 fragment significantly decreased the stiffness of isolated fCM nuclei (Fig. 7g). These data indicate that novex-3 provides elasticity/compliance to the interphase nuclei in hypoxic fCMs.

**Figure 8:**
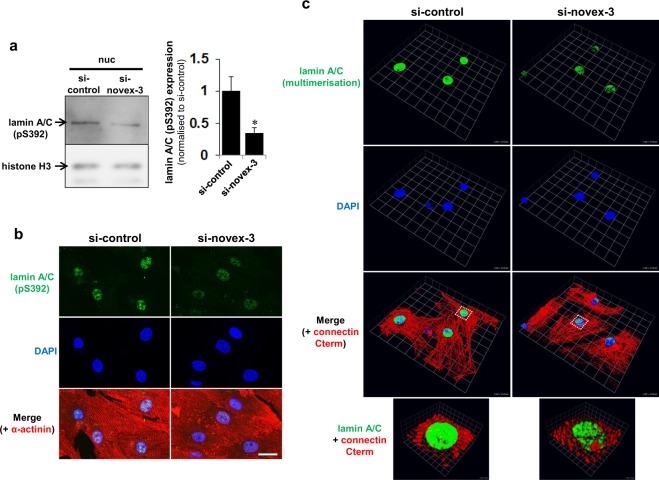
Novex-3 knockdown results in reduced phosphorylation and enhanced multimerisation of nuclear lamins in fCMs. (**a**) Immunoblot analysis for phospho-lamin A/C at Ser-392 in nuclear fraction from novex-3-silenced E17 fCMs. Histone H3 was included as a loading control. Quantitative analysis was shown on the right. n = 5 independent experiments. *P < 0.05 compared to si-control. Error bar = SEM. (**b**) Immunofluorescence staining for phospho-lamin A/C at Ser-392 (green) observed in sarcomeric α-actinin (red, as a CM marker) and DAPI (blue) in novex-3-silenced E17 fCMs. Scale bar = 20 µm. (**c**) Immunofluorescence staining for lamin A/C observed in connectin C-terminus (red, as a CM marker) and DAPI (blue) in novex-3-silenced E17 fCMs. The epitope of this lamin A/C antibody was previously shown to be buried and thus inaccessible by lamin A/C multimerisation^32,33^. Note the weak and punctate signals, and thus higher level of local lamin multimerisation in novex-3-silenced fCM nuclei. Data were shown as 3D-reconstruction images generated using Volocity. The lowest panel shows a magnified image from the boxed region in the upper panel. Scale: One unit = 13.1 µm in the upper three panels, and 1.9 µm in the lowest panel.

Generally, the two critical elements are thought to contribute to the mechanical properties of the nucleus: the lamin-based nuclear envelope structure and the DNA/histone-based chromatin structure^29^. The faint amount of novex-3 that was detectable in the chromatin-bound insoluble fraction (Fig. 1b) suggests that the latter possibility is unlikely, so we focused on lamin alterations. The lamin structure is mainly regulated by phosphorylation-dependent assembly/disassembly of higher order multimer structures, where phosphorylation favours disassembly^30,31^. Knockdown of novex-3 significantly reduced the phosphorylation level of lamin A/C in fCMs (Fig. 8a,b), suggesting an enhanced lamin multimerisation. Use of a different lamin A/C antibody, whose epitope is buried and thus inaccessible by lamin A/C multimerisation^32,33^, confirmed the higher level of multimerisation following novex-3 knockdown (Fig. 8c). These data suggest that novex-3 provides elasticity/compliance to the fCM nuclei by promoting a phosphorylation-induced disassembly of lamin multimer structures.

## Discussion

Current views on the function of novex-3 include its involvement in an elastic Z-disk to I-band linking system^20^ and in developmental myogenesis^21^, but its precise function remains elusive. In the present study, the finding that novex-3 is localised in the nucleus was unexpected and indicates a previously unrecognised role of novex-3 in promoting the proliferation of CMs specifically at the hypoxic foetal stage before birth. A nuclear function of connectin homologues has been reported in various non-muscle cells^7,10,11,14,16,17^, but this is the first report in muscle cells.

Clear nuclear staining was observed in mouse fCMs by immunofluorescence using antibodies against a novex-3-specific exon (Fig. 3a). By contrast this nuclear signal was barely detected in postnatal and adult CMs (Fig. 3a). Even in foetal CMs, no nuclear signal was detected when cultured under normoxic conditions (Fig. 4a,b). These findings explain why the nuclear novex-3 escaped detection in previous studies. Immunoblotting confirmed that the full size novex-3 (650 kDa) was expressed in the nuclear fraction (Fig. 1a,b), thereby excluding the possibility of immunofluorescent staining of short fragments or degradation products. Furthermore, exogenous expression of the N-terminal quarter of novex-3 resulted in clear nuclear localisation (Fig. 2). (The full size novex-3 (17,000 bp) is too large to be exogenously expressed at present.)

These findings raise the question of a possible role for novex-3 in the nucleus of muscle cells. In non-muscle cells, nuclear connectin homologues have been proposed to provide elasticity to nuclear structures, as connectin does for the sarcomere structure in muscles. The current literature indicates two possible functions for nuclear connectin: (1) providing elasticity to the interphase nuclei by associating with lamin filaments at the nuclear envelope and nucleoplasm^7^, and (2) providing elasticity to chromosomes, centrioles, spindles and the spindle matrix during mitosis^7,10,11,14^. In the fCMs, novex-3 was clearly found in interphase nuclei, but it was not detected in any specific structures during mitosis (Fig. 6), which suggests the likelihood of a function in interphase.

Mechanical analysis using AFM and tensile tests and biochemical analysis indicated that novex-3 provides elasticity to the interphase fCM nuclei. This possibly occurs through a promotion of phosphorylation-induced disassembly of lamin multimer structures, which results in highly deformable/compliant CM nuclei at the hypoxic foetal stage (Figs 7 and 8). The mechanism underlying the novex-3 regulation of lamin phosphorylation needs to be determined, but several studies have suggested an association of lamins with connectins or connectin homologues. In the *C. elegans* embryo, two connectin homologues, TTN-1/Ce-titin and kettin, colocalise with lamin in the interphase nuclei^7^. Similarly, the C-terminal region of human connectin interacts with lamins, and this interaction contributes to the structural integrity of the interphase nuclei^7^. Human subjects with double heterozygotic mutations of lamin A/C and connectin exhibit a more severe phenotype of dilated cardiomyopathy, with abnormally enlarged and clustered CM nuclei, when compared with subjects with a single heterozygotic lamin A/C mutation^34^.

Certain isoforms of obscurin, the only known interacting partner of novex-3, have been reported to show similar expression patterns to those of novex-3 in rat CMs (i.e. nuclear localisation at early embryonic stages and subsequent redistribution to the sarcomere during differentiation)^35^. The novex-3/obscurin complex has been proposed to have elastic properties^20,36^, so it may also contribute to the elasticity of fCM nuclei.

Cells with highly deformable nuclei are thought to proliferate actively, although the underlying mechanism remains unclear^28^. The nuclei in undifferentiated stem cells are highly deformable, but they stiffen 6-fold during terminal differentiation^37^. Cancer cells with high proliferative capacity have highly elastic/compliant nuclei^28^. We propose that novex-3 provides elasticity/compliance to the interphase CM nuclei at the hypoxic foetal stage, thereby facilitating cell cycle progression, and that the loss of novex-3 at the normoxic postnatal and adult stages results in stiffer nuclei and subsequent cell cycle arrest.

The postnatal loss of nuclear novex-3 is likely to be induced by the O_2_ elevation occurring with the onset of breathing at birth (Figs 3 and 4), and this possibility is supported by the reduced elasticity of fCM nuclei upon exposure to elevated O_2_ (Fig. 7c,f). The critical impact of O_2_ elevation at birth on CM cell cycle arrest is well recognised^1,2^. We have recently identified Fam64a as an essential molecule for activating hypoxic fCM proliferation, as it acts on the cell cycle machinery^2^. Interestingly, novex-3 knockdown inhibited Fam64a expression, and vice versa (Supplementary Fig. S7). This suggests a functional correlation between nuclear elasticity and cell cycle activity, and this may be one of the mechanisms by which cells with highly deformable nuclei undergo active proliferation.

An important open question is the nature of the regulatory mechanism that controls the different localisations of novex-3 (i.e., nucleus vs. sarcomere), given that novex-3 has an N-terminal NLS that is shared by all connectin isoforms. A broader question is why the connectin molecule, which is generally targeted toward the sarcomere, has an NLS. Machado *et al*. proposed that the nuclear and sarcomeric isoforms of *Drosophila* D-titin are distinct splice variants of the same gene, but the nuclear form lacks the N-terminal Z-repeats that binds to α-actinin in the Z-disk^10^. The expression of the seven copies of Z-repeats is known to show different copy numbers (2 to 7) in different muscles in different species, depending on the thickness of the Z-disk^38–40^. This raises the possibility that, in addition to the dependence on Z-disk thickness, another independent regulatory mechanism exists in which the isoform with fewer Z-repeats has less interaction with Z-disk and is thereby favoured for nuclear localisation via the NLS.

Notably, the rabbit heart expresses fewer copy numbers (4) at the foetal stage than at the adult stage, when the full 7 copies are expressed^39^. We found similar developmental regulation in mouse hearts (Supplementary Fig. S8), as foetal hearts expressed various isoforms containing 4 to 7 Z-repeats, which may correspond to the dual expression in the nuclei and the sarcomere at this stage. After birth, the isoforms with 4 Z-repeats progressively declined, whereas those with a larger number of Z-repeats retained their expression after birth. Discovery of an isoform lacking the entire cohort of Z-repeats, as observed in *Drosophila* D-titin, would be intriguing. Since the Z-repeats and the NLS are located in close proximity (Fig. 2a), one possibility is that the entire configuration of N-terminal region of novex-3 (and thus, of the full connectin), including the balance between the number of Z-repeats and the activity of NLS, could determine the final destination of the molecule.

This study did not fully exclude the contribution of sarcomeric novex-3 because the use of siRNAs silences both nuclear and sarcomeric novex-3. The identification of factors that regulate the splicing of seven copies of the Z-repeats and the NLS would enable the desired trafficking of the novex-3 protein to either the nucleus or the cytoplasm, thus dissecting the specific role of nuclear novex-3.

Novex-3 is well conserved among vertebrates, including humans, mice, cows, chickens, frogs (*Xenopus laevis*) and zebrafish (Supplementary Fig. S9)^21,40,41^. The reason why such a large exon (i.e., the novex-3-specific exon) has been evolutionarily conserved in the connectin gene, even though it is not utilised in the major connectin isoform, has been a longstanding mystery. The finding that novex-3 functions in the nucleus was reminiscent of invertebrate kettin, a connectin homologue found in *Drosophila* and *C. elegans*. The functioning of connectin as a nuclear protein might therefore have originated prior to the divergence of vertebrates. A more detailed discussion of this issue is provided in the Supplementary Discussion and Supplementary Figs S9 and S10.

In summary, this is the first report to indicate that nuclear connectin functions in muscle cells. Novex-3 in CM nuclei has a previously unrecognised role in promoting proliferation specifically at the hypoxic foetal stage before birth in mice, and it appears to do this by regulating lamin structures to provide the elasticity/compliance to the interphase nuclei. Identifying the molecular mechanisms regulating nuclear vs. sarcomeric transport of novex-3 and uncovering the evolutionary significance of nuclear connectins remain important challenges. Future research should therefore aim to test whether the reintroduction of novex-3 could yield elastic nuclei and activate the cell cycle machinery to drive CM proliferation/regeneration in adult hearts.

## Methods

All animal procedures were approved by the Institutional Animal Care and Use Committee at the Kawasaki Medical School. All experiments were performed in accordance with relevant guidelines and regulations of Kawasaki Medical School.

### Mi**c**e

Foetal, neonatal, and adult mice bred from a C57BL/6 background were used.

### Novex-3 antibody

In this study, we used two different novex-3 antibodies. The epitope of each antibody is depicted as “e1” and “e2” in Fig. 2a, both of which is within the novex-3-specific exon. The e1 antibody was purchased from Myomedix (Germany). The e2 antibody was raised against a synthetic peptide corresponding to residues 5113–5131 of mouse novex-3 (TPNEAIEPKDNEMPPSFIE) (Sigma). In most experiments, the e1 antibody was used, except for Fig. 4b and Supplementary Fig. S4 where the e2 antibody was used.

### Isolation and culture of primary CMs

Primary CMs were isolated from ventricles of foetal mice bred on a C57BL/6 background (embryonic day E15–E19), essentially as described^2^. Briefly, pregnant mice were euthanised with Sevofrane, and foetal heart ventricles were rapidly excised, cut into small pieces and digested four times with 0.06% trypsin and 0.24 mM EDTA in PBS for 10 min at 37 °C. After a 30 min culture to exclude non-CMs, cells were plated onto fibronectin-coated or non-coated culture vessels in DMEM with 5% FBS and cultured under the standard condition at 37 °C with 5% CO_2_. For some experiments, CMs were cultured under different O_2_ conditions (3–21%) in a multi-gas incubator (Astec, Japan).

### Baculovirus-mediated protein expression

We have previously established a baculovirus-mediated protein expression system in CMs^2^. In this study, four fragments covering the full length novex-3 (novex-3-1 through novex-3–4; Fig. 2a) and the shorter fragment containing connectin exons 1 to 6 (novex-3-Nterm; Fig. 2a) from mouse heart cDNA were independently expressed as N-terminal EGFP-tagged proteins. Full length sarcomeric α-actinin amplified from mouse heart cDNA was similarly expressed as an N-terminal mCherry-tagged protein. Baculovirus was produced in Sf9 cells, as per the manufacturer’s instructions (Thermo-Fisher). For transduction to fCMs, virus was added to the cells in MEM without serum. After 8–24 h, the medium was replaced with MEM containing 10% FBS.

### Time-lapse imaging analysis

Isolated fCMs expressing novex-3-Nterm-GFP and sarcomeric α-actinin-mCherry were placed on the stage of an inverted fluorescence microscope (All-in-One Fluorescence Microscope; Keyence, Japan) equipped with a stage incubation system. The molecular dynamics of both proteins during fCM cell division were recorded at 28 min intervals by time-lapse imaging. In separate experiments, time-lapse images were similarly obtained with the microscopic live cell analyser (JuLI Stage; NanoEnTek, Korea) to quantify the rate of fCM cell division. Complete fCM cell division events, in which mitosis was followed by cytokinesis and resulted in the generation of two daughter cells (an example shown in Fig. 6a), were manually counted and presented as the percentage of total fCMs.

### Gene silencing by siRNA

The siRNA-mediated knockdown of a specific gene was performed in cultured fCMs using Lipofectamine^®^ RNAiMAX (Thermo-Fisher). Cell density, the timing of knockdown, the amount of siRNA (mostly 15–30 nmol/L) and the timing of evaluation were optimised in each experiment. Two siRNAs against novex-3, designated as si-1 and si-2, were designed by the use of GeneAssist™ siRNA Workflow Builder (Applied Biosystems). The target sequences for both siRNAs were within novex-3-specific region of the connectin gene. The target sequences are (in the sense orientation): 5′-GCAAAUGUAUUCUCACAUA-3′ (si-1) and 5′-CCUGUUACAUUUGACCUAA-3′ (si-2). The siRNA for Fam64a was purchased from Ambion-Thermo-Fisher (Silencer Select #s99452). Corresponding negative control siRNAs were used in each experiment.

### Immunofluorescence

For tissue analyses, dissected heart tissues were fixed overnight in 4% paraformaldehyde at 4 °C, sunk in a graded series of sucrose solutions (10, 20 and 30%), and embedded in OCT compound (Tissue-Tek^®^; Sakura). They were then cut into 8 µm sections with a cryostat (Leica), permeabilised, blocked with Blocking-One (Nacalai Tesque, Japan) and labelled with primary antibodies for novex-3 (Myomedix), followed by fluorochrome-conjugated secondary antibodies. Counterstaining for DAPI and phalloidin was also performed. Essentially the same staining protocol was applied for cultured cells and isolated nuclei, except that fixation was done with methanol/acetone for some epitopes instead of 4% paraformaldehyde. The fCM nuclei were isolated using a Nuclei EZ Prep Nuclei Isolation Kit (Sigma). Primary antibodies were against novex-3 (e1 from Myomedix and e2 raised in our laboratory), Ki67 (clone SP6; Abcam), phospho-histone H3 at Ser-10 (EMD Millipore), sarcomeric α-actinin (Sigma), lamin A (Abcam), lamin A/C (clone 131C3; Abcam), phospho-lamin A/C at Ser-392 (Abcam) and connectin C-terminus (Myomedix). Samples covered with fluorescence mounting medium were examined using a confocal laser scanning system mounted on an IX81 inverted microscope (Olympus), as previously described^2^. Three-dimensional reconstruction of the z-stack images was performed using Volocity (Version 6.1.1, PerkinElmer).

### Subcellular fractionation and immunoblotting

Heart tissues were collected from mice and snap frozen in liquid nitrogen, minced and homogenised using a Kinematica™ Polytron™ homogeniser (PT1600E; Fisher Scientific) or a Micro Smash™ homogeniser (MS-100R; Tomy, Japan). For cultured CMs, harvested cell pellets were processed similarly as for heart tissues, but without the use of the homogeniser. Subcellular fractionation of proteins was done using the NE-PER system (Thermo-Fisher), which uses CER buffer for cytoplasmic extraction and NER buffer for subsequent nuclear extraction. In some samples, CER buffer was replaced with MPER buffer (Thermo-Fisher), a similar buffer designed for soluble protein extraction, to validate the results of different fractionation methods. For detailed fractionation analysis, a subcellular protein fractionation kit (Thermo-Fisher) was used, which fractionates the sample into cytoplasmic, membrane, soluble nuclear (sNuc), insoluble chromatin-bound nuclear (iNuc) and cytoskeletal fractions. Whole protein extracts without fractionation were obtained with urea-containing buffer (8 M urea, 2 M thiourea, 3% SDS, 75 mM DTT, 0.03% bromophenol blue and 0.05 M Tris-HCl pH 6.8)^42^. All of these fractionation methods were validated by immunoblotting a specific protein with known localisation (Fig. 1c,d): β-tubulin (cytoplasmic), nup98 (nuclear), fibrillarin (nuclear), rab5 (membrane) and vimentin (cytoskeletal). The antibodies for these five proteins were obtained from Cell Signaling. The antibodies for phospho-lamin A/C at Ser-392 (Abcam), GFP (Santa Cruz), and histone H3 (Cell Signaling) were also used. All extraction procedures included a protease inhibitor cocktail. Efficient detection of the novex-3 protein, which is quite large in size (650 kDa), was ensured by applying the following conditions (modified from ref.^42^): Protein samples were heated at 60 °C for 10 min and separated by SDS-PAGE on a 4–15% gradient polyacrylamide gel (Mini-PROTEAN® TGX; Bio-Rad) at 10–20 mA constant for 2–3 h with constant cooling. The composition of the lower electrophoresis buffer was 50 mM Tris, 384 mM glycine and 0.1% SDS. The upper buffer contained the same with the addition of 10 mM β-mercaptoethanol. A standard for high molecular weight proteins (up to 460 kDa) was included (HiMark™ Pre-Stained Protein Standard, Thermo-Fisher). The samples were then transferred onto PVDF membranes in the transfer buffer (25 mM Tris, 192 mM glycine and 20% methanol supplemented with 10 mM β-mercaptoethanol) at 40 V constant for 4–5 h with constant cooling. The membranes were blocked with 3% BSA or 3% nonfat milk in TBS/T, probed with primary antibodies for novex-3 (Myomedix) followed by secondary horseradish peroxidase (HRP)-conjugated IgG and finally visualised by enhanced chemiluminescence (Western Lightning ECL-Pro; PerkinElmer) using a LAS4000mini luminescent image analyser (GE Healthcare), as previously described^43^.

### **Quantitative PCR (qPCR)**

Hearts were collected from mice, cut into small pieces and immediately immersed in RNAlater® Stabilization Reagent (QIAGEN). The stabilised tissues were homogenised with a Micro Smash™ homogeniser, and total RNA was isolated using ISOGEN system (Nippon Gene, Japan). For cultured CMs, harvested cell pellets were processed similarly as for heart tissues, but without the use of the homogeniser. After assessing RNA yield and quality using a NanoDrop spectrophotometer (ND-1000; Thermo-Fisher), the RNA samples were reverse-transcribed with PrimeScrip RT Master Mix (TaKaRa Bio), and quantitative real-time PCR was performed using TaqMan® Fast Advanced Master Mix in a StepOnePlus™ real-time PCR system (Applied Biosystems). The TaqMan® probe specific for novex-3 was designed using the Custom Taqman Assay Design Tool (Applied Biosystems). The target sequence for the probe was within the novex-3-specific region of the connectin gene, which is not disclosed due to the regulations of the manufacturer. Other TaqMan^®^ gene expression assays used were for *Ccna2* (#Mm00438063_m1), *Ccne2* (#Mm00438077_m1), *Egln3* (#Mm00472200_m1), *Aurkb* (#Mm01718146_g1), *Cdca5* (#Mm01233533_m1), *Fam64a* (#Mm01245821_g1) and *EGFP* (Mr04097229_mr). Quantification of each mRNA was carried out with *Actb* (#Mm00607939_s1), *Rn18S* (#Mm03928990_g1), or *18S* (#Hs99999901_s1) as reference genes, using the ΔΔC_T_ method.

### Atomic force microscopy (AFM)

The mechanical properties of fCMs were evaluated with AFM indentation measurements (NVB100; Olympus), which we have previously established for various cell types including CMs^23,24,27^. The AFM unit was mounted on an inverted fluorescent microscope. Briefly, intrusion of the AFM cantilever onto the cell generated a force-indentation curve, which is the relationship between the cantilever deflection and its indentation depth onto the cell (Fig. 7a). The elastic modulus (*E*) was calculated based on the Hertz model, which describes the indentation of a homogeneous/semi-infinite elastic material^44^, defined as follows:

$$F={\delta }^{2}\frac{\pi }{2}\frac{E}{(1-{\nu }^{2})}\,\tan \,\alpha $$

where *F* is the applied nano-order force (calculated from the spring constant of the cantilever [0.08 N/m] multiplied by the cantilever deflection), *E* is the elastic modulus, *ν* is the Poisson’s ratio (assumed to be 0.5 as the cell was considered incompressible), *α* is the opening angle of the tip of the cantilever and *δ* is the micro-order indentation depth. The cantilever was calibrated before each experiment. Measurements were performed on the central region of the nuclei, as monitored by phase contract optics, as previously described^26–28,33^. Cells showing signs of mitosis were excluded. The experiments were carried out at room temperature.

### Microneedle-based tensile test of a single isolated fCM nucleus

The fCM nuclei were isolated using Nuclei EZ Prep Nuclei Isolation Kit (Sigma) from an fCM culture isolated from foetal hearts (E17–E18). The integrity of the isolated nuclei was verified by DAPI and lamin staining (Supplementary Fig. S2). Microneedle-based tensile tests were performed based on the published literature^29^, and the experimental set up was slightly modified from a laboratory-made tensile test system consisting of an inverted microscope (IX-73; Olympus), a 40x water immersion objective lens (UAPON40XW340; Olympus), a digital CMOS camera (ORCA flash 4.0; Hamamatsu Photonics) and a pair of glass microneedles each connected to three-axis micromanipulators^45^. One microneedle, designated as an operation microneedle, was rigid and was moved with a three-axis motorised micromanipulator (EMM2; Narishige, Japan) to stretch the nucleus. The other microneedle, designated as a deflection microneedle, was flexible, to obtain the force applied to the nucleus by measuring its deflection. The spring constant of the microneedle was determined by a cross-calibration method^46,47^. A single isolated nucleus was captured by putting the microneedle tips into the nucleus (Fig. 7d). The nucleus was then lifted off the chamber bottom and stretched horizontally at a rate of 1 μm/s by moving the operation microneedle along the surface of the chamber bottom. The experiments were carried out at room temperature. The positions of the tips of microneedles were measured using MetaMorph Offline software (version 7.7.0.0; Molecular Devices). The distance between the tips of the two microneedles along the axis of stretch was defined as *L*. The nuclear deformation, *D*, was defined as the difference between *L* and the initial distance *L*_0_. The force, *F*, applied to the nucleus was calculated by multiplying the deflection of the deflection microneedle, *X*, by its spring constant. The stiffness was defined as the slope of the force (*F*)-deformation (*D*) curve within the deformation range of 0–2 μm, based on the assumption that the curve is piecewise linear.

### Statistics

All data were expressed as mean plus or minus standard error of the mean (SEM). For comparisons between two groups, Student’s two-tailed paired t-test was used to determine statistical significance. For comparisons among multiple groups, one-way analysis of variance (ANOVA) was used with Bonferroni’s post hoc test. P < 0.05 was considered statistically significant. Significance levels were indicated as *P < 0.05, **P < 0.01, and ***P < 0.001.

## Electronic supplementary material

Supplementary Information

Supplementary Movie S1

Supplementary Movie S2

Supplementary Movie S3

## Data Availability

All data generated or analysed during this study are included in this published article (and its Supplementary Information files).
