# Supplementary material for: Nuclear connectin novex-3 promotes proliferation of hypoxic foetal cardiomyocytes
Source: Sci Rep. 2018 Aug 17;8:12337. doi: 10.1038/s41598-018-30886-9 (PMC6098106; doi:10.1038/s41598-018-30886-9)

# Nuclear connectin novex-3 promotes proliferation of hypoxic foetal cardiomyocytes.

*Ken Hashimoto, Aya Kodama, Miki Sugino, Tomoko Yobimoto, Takeshi Honda, Akira Hanashima, Yoshihiro Ujihara, and Satoshi Mohri

# Supplementary Figures S1-S10

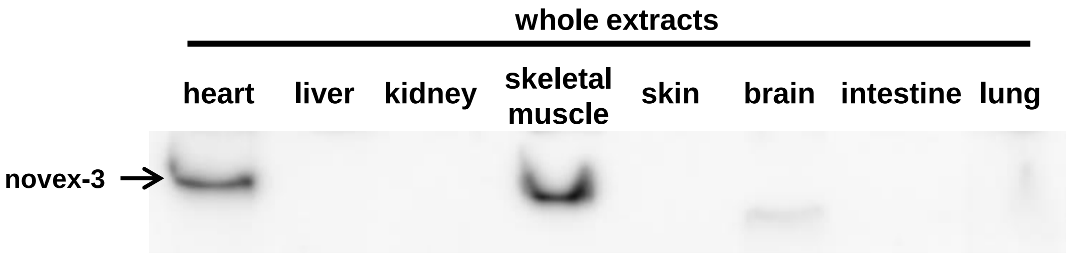


**Supplementary Fig. S1. Striated muscle-restricted expression of novex-3 protein.** Immunoblot analysis for novex-3 in whole protein extracts from various mouse tissues.

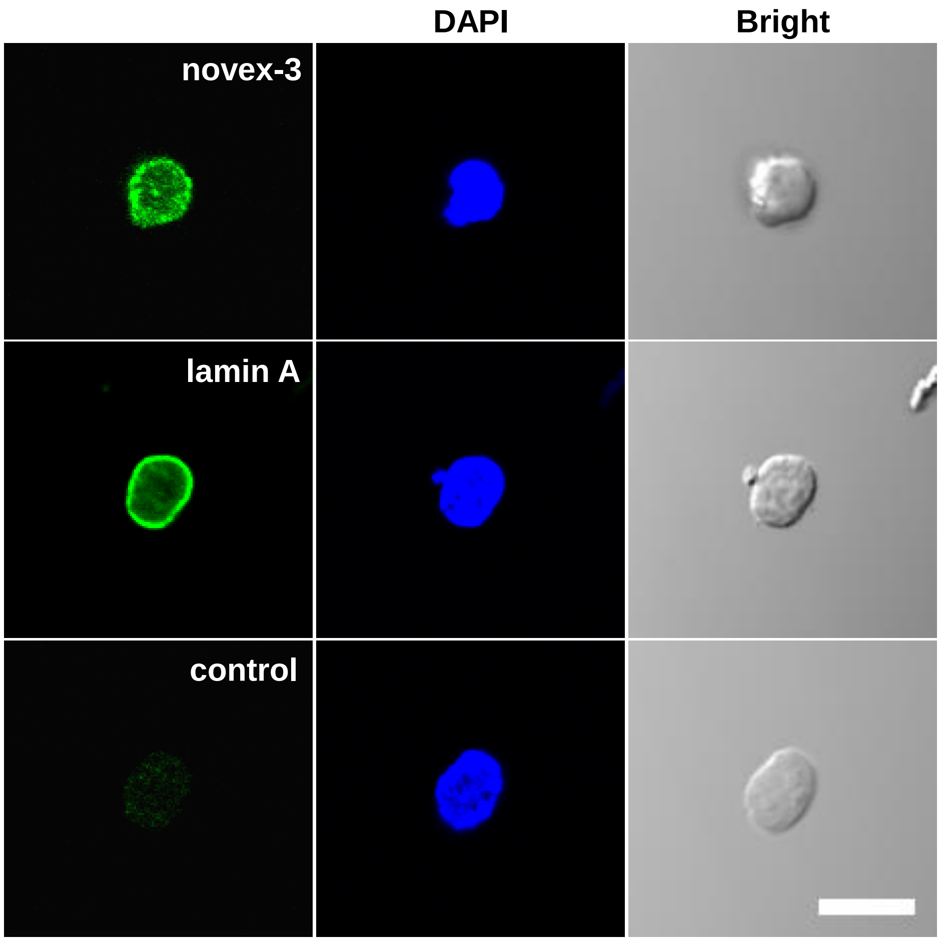


**Supplementary Fig. S2. Novex-3 protein is detected in isolated fCM nuclei.**
The expression of novex-3 protein was confirmed by immunostaining of the isolated E17 fCM nuclei preparation. The integrity of the isolated nuclei was verified by lamin staining, DAPI staining, and bright-field images. Negative control samples without the primary antibody were also shown. Scale bar = 10µm.

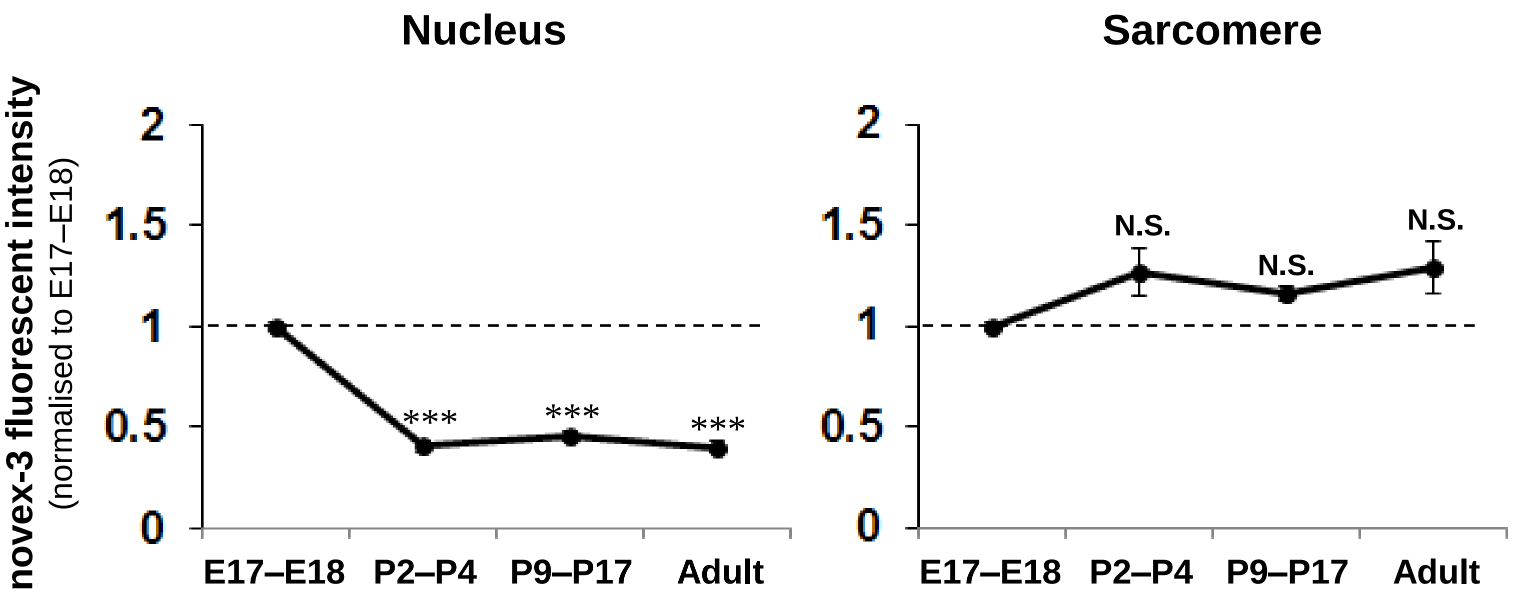


**Supplementary Fig. S3. The nuclear expression of novex-3 is repressed after birth, whereas the sarcomeric expression remains unchanged.**

Novex-3 immunofluorescence was quantified separately in the nucleus and the sarcomere at indicated stages using Image J. The regions of the nucleus and the sarcomere were identified by DAPI and phalloidin staining, respectively, from the immunostaining image (an example is shown in Fig. 3a). The sarcomeric region was roughly determined to be the intracellular region except the nucleus (i.e., the cytoplasm). n = 22–28 cardiomyocytes from 2–3 different hearts. ***P < 0.001 compared to E17–E18 by one-way ANOVA. N.S.: not significant compared to E17–E18 by one-way ANOVA. Error bars = SEM. E: embryonic day, P: postnatal day.

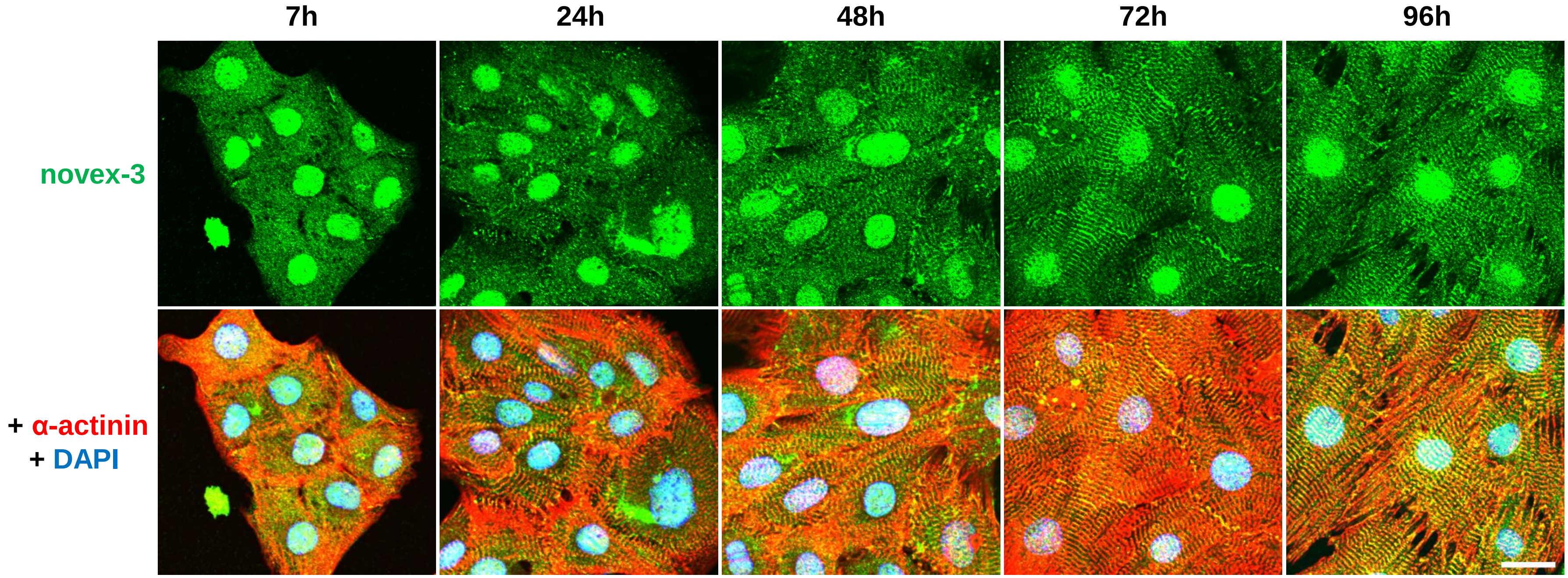


**Supplementary Fig. S4. In vitro culture itself did not affect the expression pattern of novex-3 in fCMs.**
Immunofluorescence staining for novex-3 (green) observed in sarcomeric α-actinin (red, as a CM marker) and DAPI (blue) in E15–17 fCMs cultured under 3% $O_2$ conditions. Strong nuclear expression of novex-3 was observed 7 h after plating, when fCMs started to attach to the culture plate, whereas sarcomeric expression was rarely observed at this stage. During subsequent culture, cells spread on the surface of the culture plate and redeveloped sarcomere structure, which had been transiently disrupted by enzymatic cell isolation. The expression pattern of novex-3 in intact fCMs—slight sarcomeric expression and strong nuclear expression (as shown in Fig. 3a)—was finally recapitulated at 48 h or later. Scale bar = 20 µm.

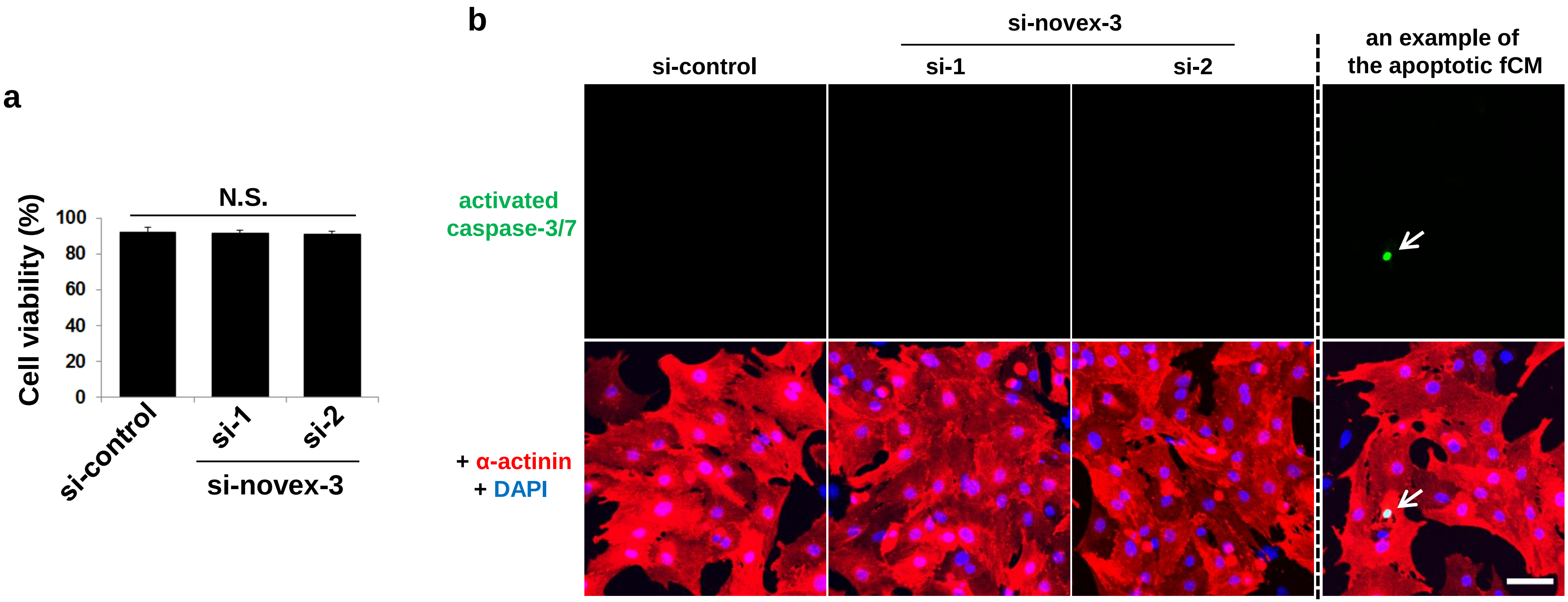


**Supplementary Fig. S5. Validation of siRNAs targeting novex-3 used in this study.**

a. fCMs were treated with the indicated siRNA, and cell viability was assessed by trypan blue exclusion. n = 3 independent experiments.

b. fCMs were treated with the indicated siRNA, and apoptotic cells were detected using CellEvent Caspase-3/7 Green Detection Reagent, as per the manufacturer's instructions (Thermo-Fisher). After fixation, cells were immunostained for sarcomeric α-actinin (as a CM marker) and DAPI. No apoptotic fCMs were detected in any sample/condition in 3 independent experiments. Arrows denote the rare apoptotic fCM showing bright nuclear fluorescence which is generated by activated caspase-3/7 in this assay. Scale bar = 50 µm.

The data indicate that these siRNAs (si-1 and si-2) have no toxicity or apoptosis-inducing effects and thus are valid for use in functional studies. E15–E17 fCMs were used. N.S.: not significant among groups by one-way ANOVA. Error bars = SEM.

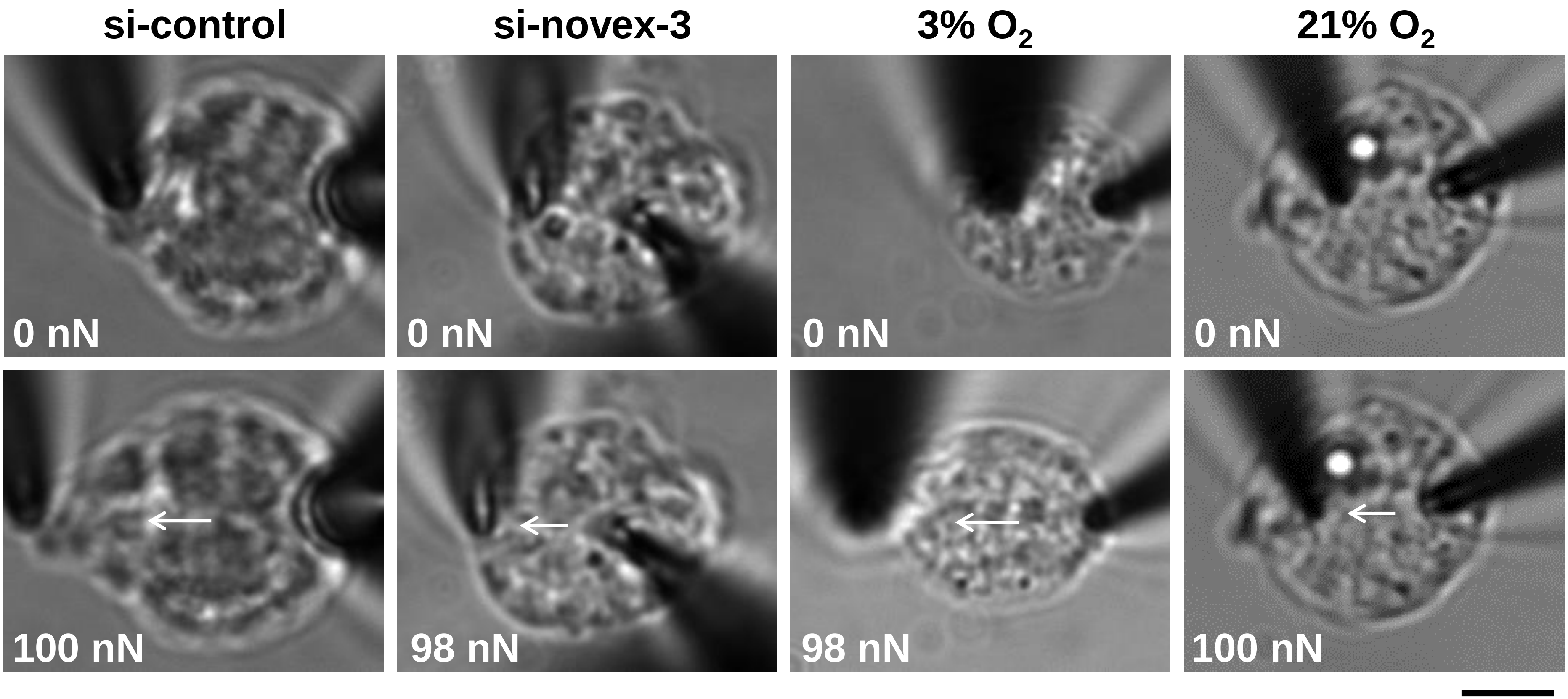


**Supplementary Fig. S6. Microneedle-based tensile tests of a single isolated fCM nucleus.** Representative images of a single isolated fCM nucleus (E17–E18) before (upper panel) and during (lower panel) stretch in each experimental condition. Lower panels show the image when the force of about 100 nN was applied to the nucleus. Arrows, direction of stretch. Scale bar = 5µm.

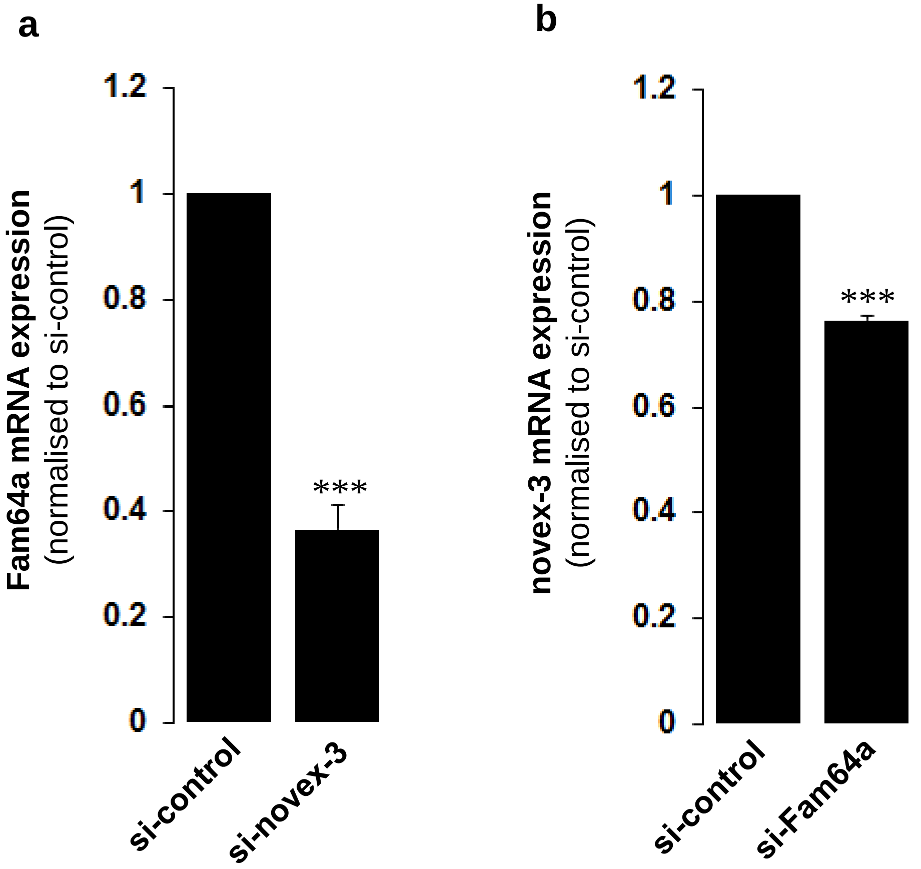


**Supplementary Fig. S7. Functional correlation between novex-3 and Fam64a.**

**a.** qPCR analysis of Fam64a mRNA expression in novex-3-silenced E16–E18 fCMs. n = 3 independent experiments. ***P < 0.001 compared to si-control. Error bar = SEM.

**b.** qPCR analysis of novex-3 mRNA expression in Fam64a-silenced E16–E18 fCMs. n = 3 independent experiments. ***P < 0.001 compared to si-control. Error bar = SEM.

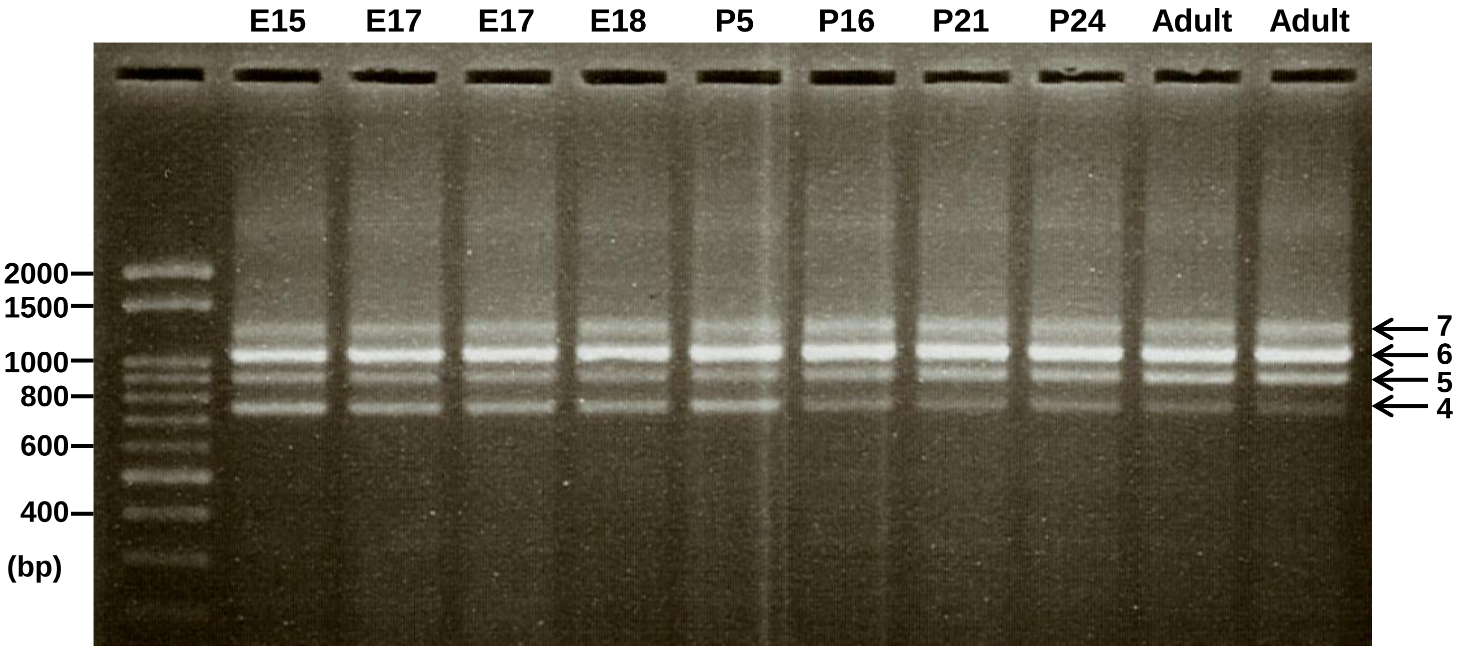


**Supplementary Fig. S8. Developmental regulation of Z-repeats expression in mouse hearts.** cDNAs from mouse hearts at indicated stages were PCR amplified using primers flanking the entire seven copies of Z-repeats in the connecin gene. PCR products were electrophoresed in 2% agarose gels and visualized. The number shown on the right (4 to 7) indicates the number of Z-repeats expressed in each band, that was confirmed by sequencing. E; embryonic day, P; postnatal day.

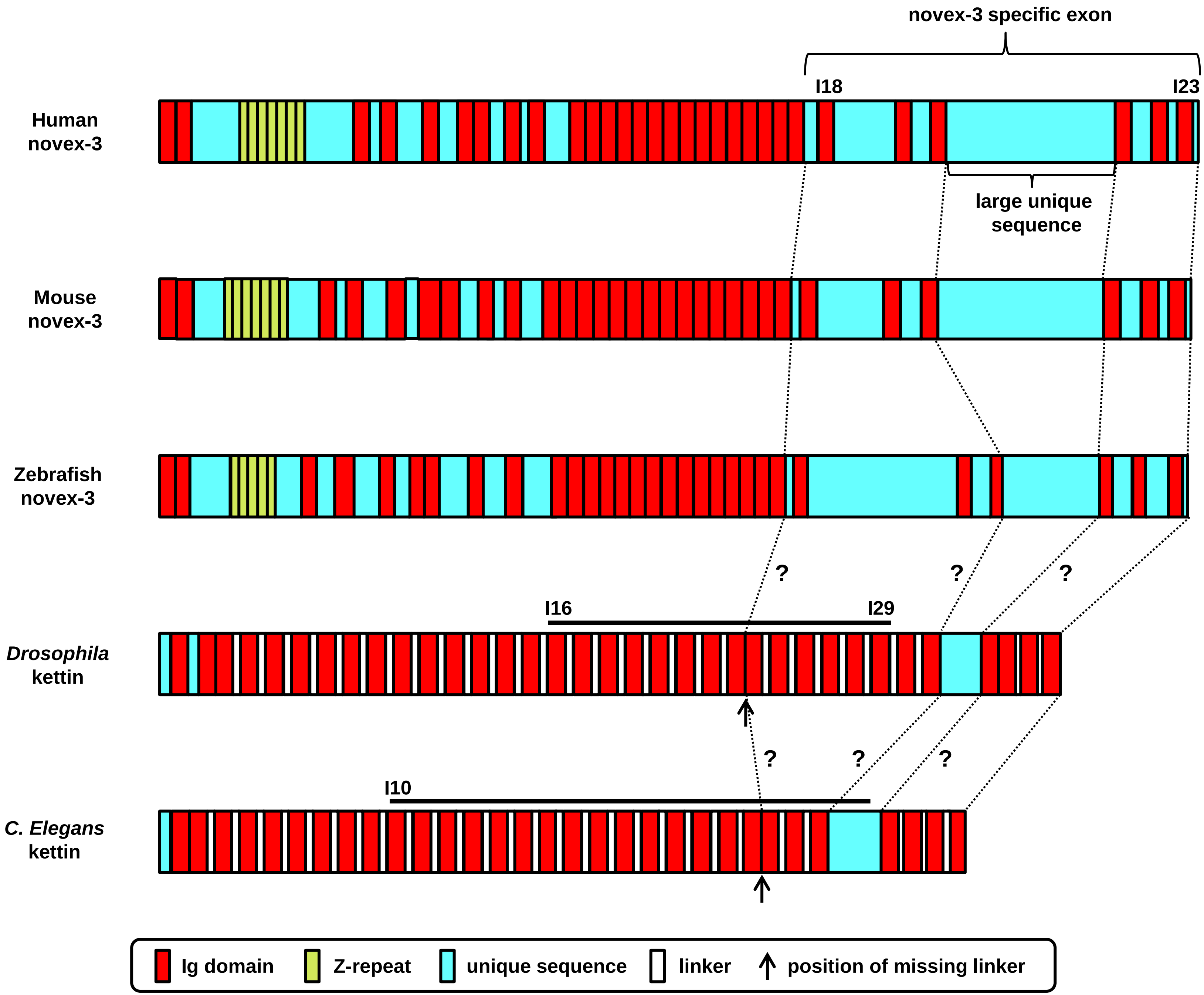
novex-3 specific exon
I18
I23
Human novex-3
large unique sequence
Mouse novex-3
Zebrafish novex-3
?
?
?
I16
I29
Drosophila kettin
?
?
?
I10
C. Elegans kettin
Ig domain
Z-repeat
unique sequence
linker
position of missing linker

**Supplementary Fig. S9. Evolutionary conservation among vertebrate novex-3 (humans, mice, and zebrafish) and invertebrate kettin (*Drosophila* and *C.elegans*).**

Primary structure of vertebrate novex-3 (humans, mice, and zebrafish) and invertebrate kettin (*Drosophila* and *C.elegans*). Vertical dotted lines indicate the possible molecular relationships among species. Arrows in *Drosophila* and *C.elegan* kettin show the conserved position of missing linker. Horizontal lines on *Drosophila* and *C.elegan* kettin show the region corresponding to the largest exon in each molecule. See Supplementary Discussion for details. Primary structure was compiled from literature as follows; human novex-3[1,2], mouse novex-3[3], zebrafish novex-3[4,5], *Drosophila* kettin[6-8], and *C.elagans* kettin[6,7,9]. The main purpose of this figure is to compare the gross structure of each molecule among species, thus the length of each domain is approximately, but not precisely, proportional to the number of amino acids.

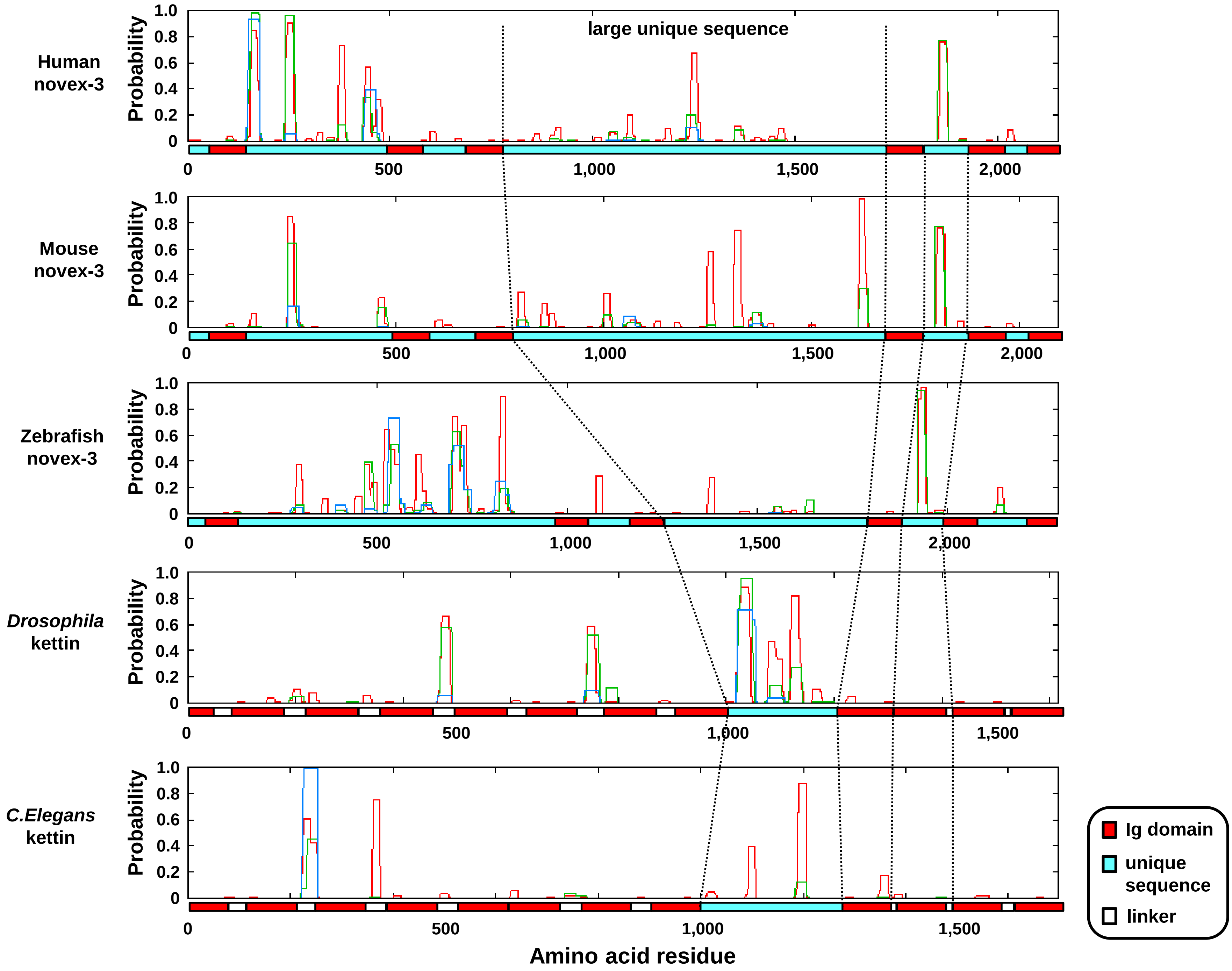

Human novex-3
Mouse novex-3
Zebrafish novex-3
*Drosophila* kettin
*C.Elegans* kettin
Probability
large unique sequence
Amino acid residue
Ig domain
unique sequence
linker

**Supplementary Fig. S10. Coiled-coil potential is conserved among vertebrate novex-3 (humans, mice, and zebrafish) and invertebrate kettin (*Drosophila* and *C.elegans*).**
Coiled-coil potential near the C-terminal region of each molecule was predicted by the Coiled-Coils program[10] . In all five species, the most downstream position in horizontal axis corresponds to the C-terminal end of each molecule. In vertebrate novex-3, the entire novex-3-specific exon (about 2000 amino acids) is covered. In invertebrate kettin, the most upstream point in horizontal axis corresponds to the position 1000 amino acids upstream of the initiation point of the large unique sequence, thus covering about 1700 amino acids. Vertical dotted lines indicate the possible molecular relationships among species. Blue, green, and red plot: the number of amino acid residues, 28, 21, and 14 respectively, used to calculate the coiled-coil potential as shown in probability (0 to 1) in the vertical axis.

## References for Supplementary Figures


**1.** Bang, M. L. *et al.* The complete gene sequence of titin, expression of an unusual approximate to 700-kDa titin isoform, and its interaction with obscurin identify a novel Z-line to I-band linking system. *Circulation Research* **89**, 1065-1072, doi:10.1161/hh2301.100981 (2001).

**2.** Kellermayer, D., Smith, J. E. 3rd. & Granzier, H. Novex-3, the tiny titin of muscle. *Biophys Rev.* **9**, 201-206, doi: 10.1007/s12551-017-0261-y (2017).

**3.** Granzier, H. *et al.* Functional genomics of chicken, mouse, and human titin supports splice diversity as an important mechanism for regulating biomechanics of striated muscle. *American Journal of Physiology-Regulatory Integrative and Comparative Physiology* **293**, R557-R567, doi:10.1152/ajpregu.00001.2007 (2007).

**4.** Seeley, M. *et al.* Depletion of zebrafish titin reduces cardiac contractility by disrupting the assembly of Z-discs and A-bands. *Circulation Research* **100**, 238-245, doi:10.1161/01.RES.0000255758.69821.b5 (2007).

**5.** Hanashima, A. *et al.* Complete primary structure of the I-band region of connectin at which mechanical property is modulated in zebrafish heart and skeletal muscle. *Gene* **596**, 19-26, doi:10.1016/j.gene.2016.10.010 (2017).

**6.** Kolmerer, B. *et al.* Sequence and expression of the kettin gene in Drosophila melanogaster and Caenorhabditis elegans. *Journal of Molecular Biology* **296**, 435-448, doi:10.1006/jmbi.1999.3461 (2000).

**7.** Bullard, B., Linke, W. A. & Leonard, K. Varieties of elastic protein in invertebrate muscles. *Journal of Muscle Research and Cell Motility* **23**, 435-447, doi:10.1023/a:1023454305437 (2002).

**8.** Bullard, B., Burkart, C., Labeit, S. & Leonard, K. The function of elastic proteins in the oscillatory contraction of insect flight muscle. *Journal of Muscle Research and Cell Motility* **26**, 479-485, doi:10.1007/s10974-005-9032-7 (2005).

**9.** Ono, K., Yu, R., Mohri, K. & Ono, S. Caenorhabditis elegans kettin, a large immunoglobulin-like repeat protein, binds to filamentous actin and provides mechanical stability to the contractile apparatuses in body wall muscle. *Molecular Biology of the Cell* **17**, 2722-2734, doi:10.1091/mbc.E06-02-0114 (2006).

**10.** Lupas, A., Van Dyke, M. & Stock, J. Predicting coiled coils from protein sequences. *Science* **252**, 1162-1164 (1991).

# Supplementary Discussion

Novex-3 is well conserved among vertebrates, including humans, mice, cows, chickens, frogs (*Xenopus laevis*) and zebrafish (Supplementary Fig. S9)[1-3]. The reason why such a large exon (i.e., the novex-3-specific exon) has been evolutionarily conserved in the connectin gene, even though it is not utilised in the major connectin isoform, has been a longstanding mystery. The finding that novex-3 functions in the nucleus was reminiscent of invertebrate kettin, a connectin homologue found in *Drosophila* and *C. elegans*. The functioning of connectin as a nuclear protein might therefore have originated prior to the divergence of vertebrates. The following is the detailed discussion regarding the similarity between novex-3 and kettin.

Kettin was initially discovered as a Z-disk "mini-connectin" in the giant waterbug *Lethocerus*[4], *Drosophila*[5] and *C. elegans*[6], which is reminiscent of novex-3. Novex-3 and kettin also have a similar molecular size (500–650 kDa), and both proteins are N-terminal truncated variants of the parent connectin gene; the exception is the *C. elegans* kettin, which is derived from a single distinct gene[7]. Kettin has been reported to localise in the nucleus under certain circumstances, whereas its major location is at the sarcomeric Z-disk[8-10], which is similar to the localisation of novex-3. The N-terminal NLS of novex-3 is well conserved among humans (200-PAKKTKT), mice (194-PAKKTKT) and zebrafish (200-PAKKSKT). In kettin, a putative NLS at a similar location was predicted by the SeqNLS program (274-KVDKVERSR)[11]. Kettin mRNA in insect larva is also upregulated 4.5-fold following 24 h of hypoxia (3% $O_2$) with hypercapnia (17% $CO_2$)[12], again similar to novex-3 (Fig. 4).

Our in silico analysis has also shown that the domain structure is, to some extent, expected to be similar between kettin and novex-3. As shown in Supplementary Fig. S9, we focused on the large novex-3-specific exon near the C-terminus. This is the largest exon (~7000 bp) in novex-3, and is the second largest exon among the ~350 exons in the whole connectin gene. This exon contains 5' three Ig domains, the large unique sequence in the middle, and 3' three Ig domains, and is well conserved among humans, mice and zebrafish, suggesting an evolutionary importance of maintaining this unit as a single exon. Although the corresponding region is difficult to determine in evolutionarily distant species, the kettin in *Drosophila* and *C. elegans* has a similar large unique sequence in front of four Ig domains near the C terminus (Supplementary Fig. S9).

# Supplementary Discussion (continued)

The size of the large unique sequence differs substantially between vertebrates (500–900 residues) and invertebrates (200–300 residues), but this is the largest unique segment in each species, apart from zebrafish, which has one larger segment upstream in the novex-3 exon. This large segment in all five species shows a conserved feature in that they contain ~30% SEK (serine (S), glutamic acid (E) and lysine (K)), the amino acid bias found in crayfish I-connectin[13]. In the downstream three Ig domains in the vertebrate novex-3, only the unique 100-amino acid segment between the first and the second Ig domains contains as high as 40% PEVK (proline (P), glutamic acid (E), valine (V), and lysine (K)), and this is conserved among humans, mice and zebrafish. In invertebrate kettin, four Ig domains are found, but only the unique (linker) segment both 5' and 3' of the second Ig domain contains equivalent or more PEVK. This suggests that one more kettin Ig domain that is not found in vertebrates is the second Ig domain, and the overall domain structure is conserved (Supplementary Figs. S9, S10). This is supported by the fact that the number of residues in the second Ig domain plus the 5' and 3' unique segment in invertebrate kettin almost equals the number of residues in the unique segment between the first and the second Ig domains in the vertebrate novex-3 (108~110 residues).

Previously, N-terminal 450 amino acid segment of the novex-3 exon in humans, mice, and chickens was predicted to form coiled-coil structures and to show the highest probability for dimer formation over the entire I-band region[14,15]. We confirmed this and further extended the analysis to cover invertebrate kettin using the Coiled-Coils program[16]. As shown in Supplementary Fig. S10, the coiled-coil-susceptible region exclusively maps to the unique or linker segment, and is frequently observed throughout the vertebrate novex-3 exon and the corresponding region in invertebrate kettin, suggesting conservation of the potential for multimer formation.

The 5' border of the kettin region that corresponds to the novex-3 exon is more difficult to consider. One possibility is that a well conserved position in kettin gene (and the parent D-titin gene) that lacks linker sequence between Ig domains[7] may be the 5' border (Supplementary Fig. S9, arrow). Alternatively, the largest exon in kettin is similar in size to the novex-3 exon (~5500 bp in *Drosophila* and ~7500 bp in *C. elegans*) and thus it may relate to the 5' border (Supplementary Fig. S9).

In summary, the data suggest that invertebrate kettin could be evolutionarily related to vertebrate novex-3. The functioning of connectin as a nuclear protein might therefore have originated prior to the divergence of vertebrates.

## References for Supplementary Discussion


**1.** Brown, D. D., Davis, A. C. & Conlon, F. L. Xtn3 is a developmentally expressed cardiac and skeletal muscle-specific novex-3 titin isoform. *Gene Expression Patterns* **6**, 913-918, doi:10.1016/j.modgep.2006.03.003 (2006).

**2.** Seeley, M. *et al.* Depletion of zebrafish titin reduces cardiac contractility by disrupting the assembly of Z-discs and A-bands. *Circulation Research* **100**, 238-245, doi:10.1161/01.RES.0000255758.69821.b5 (2007).

**3.** Hanashima, A. *et al.* Complete primary structure of the I-band region of connectin at which mechanical property is modulated in zebrafish heart and skeletal muscle. *Gene* **596**, 19-26, doi:10.1016/j.gene.2016.10.010 (2017).

**4.** Lakey, A. *et al.* Identification and localization of high molecular weight proteins in insect flight and leg muscle. *EMBO J*. **9**, 3459-3467 (1990).

**5.** Lakey, A. *et al.* KETTIN, A LARGE MODULAR PROTEIN IN THE Z-DISC OF INSECT MUSCLES. *Embo Journal* **12**, 2863-2871 (1993).

**6.** Kolmerer, B. *et al.* Sequence and expression of the kettin gene in Drosophila melanogaster and Caenorhabditis elegans. *Journal of Molecular Biology* **296**, 435-448, doi:10.1006/jmbi.1999.3461 (2000).

**7.** Bullard, B., Linke, W. A. & Leonard, K. Varieties of elastic protein in invertebrate muscles. *Journal of Muscle Research and Cell Motility* **23**, 435-447, doi:10.1023/a:1023454305437 (2002).

**8.** Flaherty, D. B. *et al.* Titins in C-elegans with unusual features: Coiled-coil domains, novel regulation of kinase activity and two new possible elastic regions. *Journal of Molecular Biology* **323**, 533-549, doi:10.1016/s0022-2836(02)00970-1 (2002).

**9.** Bullard, B., Burkart, C., Labeit, S. & Leonard, K. The function of elastic proteins in the oscillatory contraction of insect flight muscle. *Journal of Muscle Research and Cell Motility* **26**, 479-485, doi:10.1007/s10974-005-9032-7 (2005).

**10.** Zastrow, M. S., Flaherty, D. B., Benian, G. M. & Wilson, K. L. Nuclear titin interacts with A- and B-type lamins in vitro and in vivo. *Journal of Cell Science* **119**, 239-249, doi:10.1242/jcs.02728 (2006).

**11.** Lin, J. R., Mondal, A. M., Liu, R. & Hu, J. J. Minimalist ensemble algorithms for genome-wide protein localization prediction. *Bmc Bioinformatics* **13**, doi:10.1186/1471-2105-13-157 (2012).

**12.** Chi, Y. H. *et al.* Changes in oxygen and carbon dioxide environment alter gene expression of cowpea bruchids. *Journal of Insect Physiology* **57**, 220-230, doi:10.1016/j.jinsphys.2010.11.011 (2011).

**13.** Fukuzawa, A. *et al.* Invertebrate connectin spans as much as 3.5 mu m in the giant sarcomeres of crayfish claw muscle. *Embo Journal* **20**, 4826-4835, doi:10.1093/emboj/20.17.4826 (2001).

**14.** Granzier, H. *et al.* Functional genomics of chicken, mouse, and human titin supports splice diversity as an important mechanism for regulating biomechanics of striated muscle. *American Journal of Physiology-Regulatory Integrative and Comparative Physiology* **293**, R557-R567, doi:10.1152/ajpregu.00001.2007 (2007).

**15.** Kellermayer, D., Smith, J. E. 3rd. & Granzier, H. Novex-3, the tiny titin of muscle. *Biophys Rev.* **9**, 201-206, doi: 10.1007/s12551-017-0261-y (2017).

**16.** Lupas, A., Van Dyke, M. & Stock, J. Predicting coiled coils from protein sequences. *Science* **252**, 1162-1164 (1991).

# Legend for Supplementary Movies S1-S3

**Supplementary movies S1–S3. Novex-3 does not localise to any specific structures during mitosis in fCMs.**
A representative time-lapse recording of the cell division dynamics of fCMs doubly expressing novex-3-Nterm-GFP and sarcomeric α-actinin-mCherry (as a CM marker), corresponding to Fig. 6a. In contrast to the clear nuclear expression observed during interphase, novex-3-Nterm expression was not localised to specific structures (spindles, spindle matrix, centrioles, or chromosomes) during mitosis; instead, it was diffusely spread throughout the cytoplasm following nuclear envelope breakdown at prometaphase. At telophase, when the nuclear envelope reassembled, the novex-3-Nterm re-accumulated in the newly formed daughter nuclei, where it was maintained over the next interphase. The number on each panel indicates the time in (hours: minutes) elapsed from the beginning. Note that the panel at 38:16 in these movies corresponds to the panel at 0:00 (interphase) in Fig. 6a. Scale bar = 50µm. Movie S1: novex-3-Nterm-GFP channel, Movie S2: novex-3-Nterm-GFP + sarcomeric α-actinin-mCherry channel, Movie S3: novex-3-Nterm-GFP + sarcomeric α-actinin-mCherry + bright-field channel.

# Full-length blots

## Fig 1b

novex-3

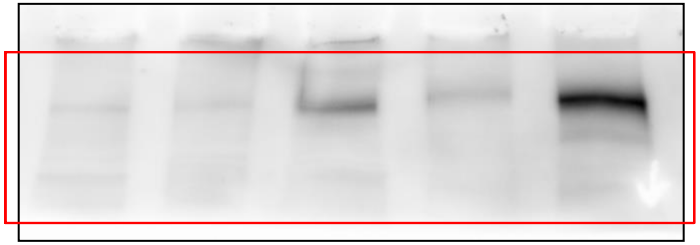

## Fig 1c

β-tubulin (CER/NER)

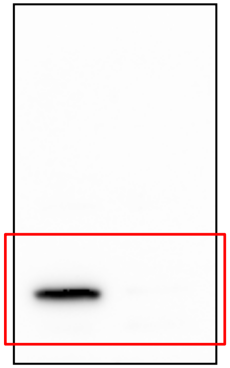

nup98 (CER/NER)

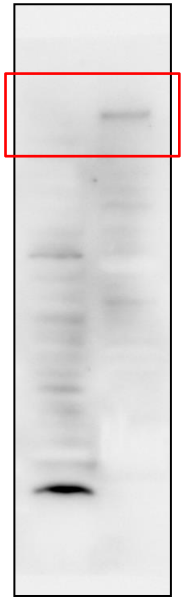

fibrillarin (CER/NER)

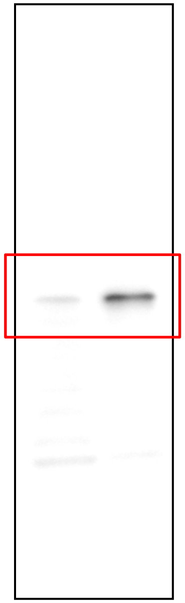

β-tubulin (MPER/NER)

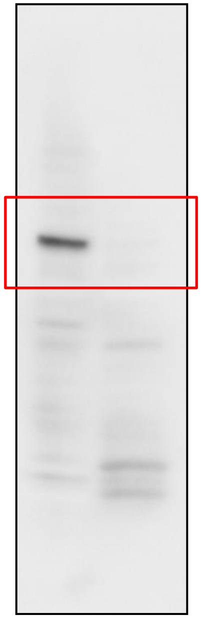

nup98 (MPER/NER)

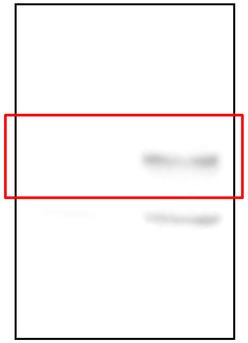

fibrillarin (MPER/NER)

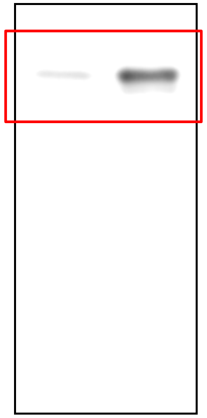

**Fig 1d**

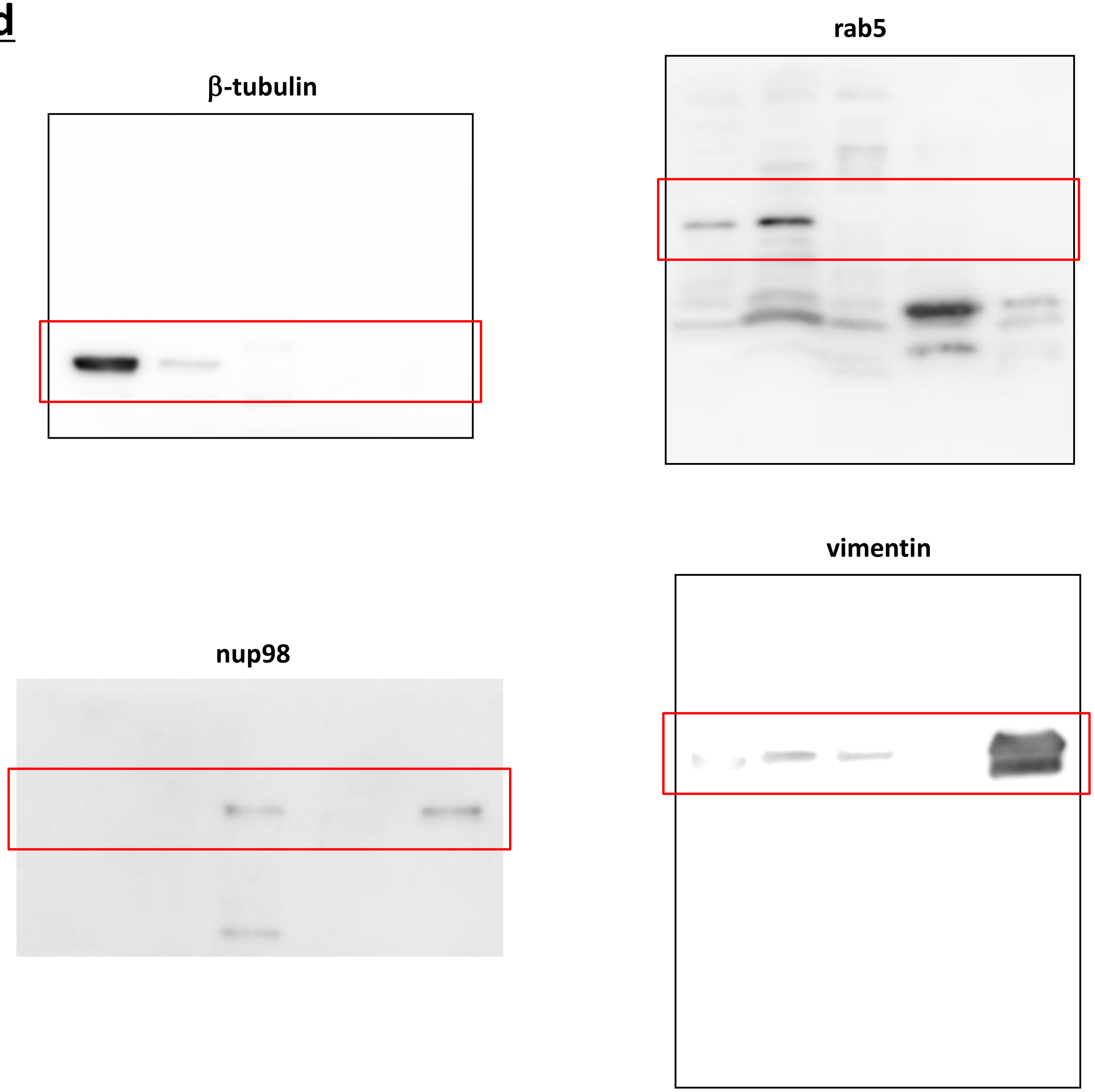

# Fig 3c

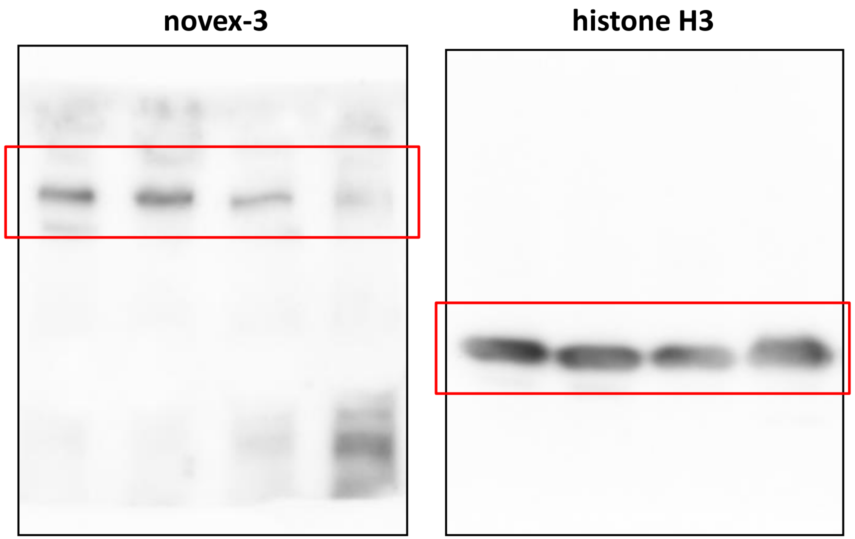


# Fig 4d

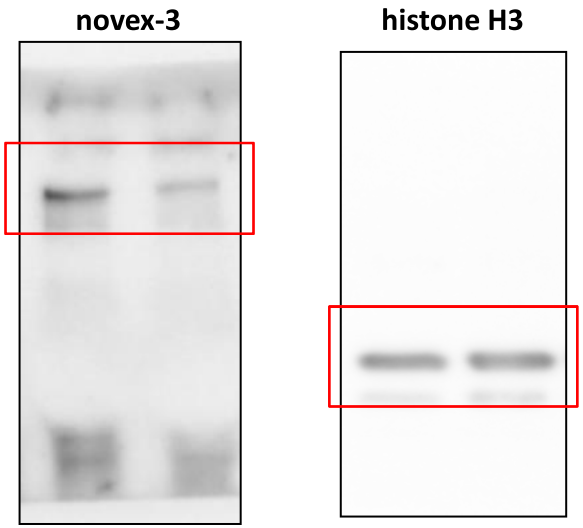


# Fig 5a

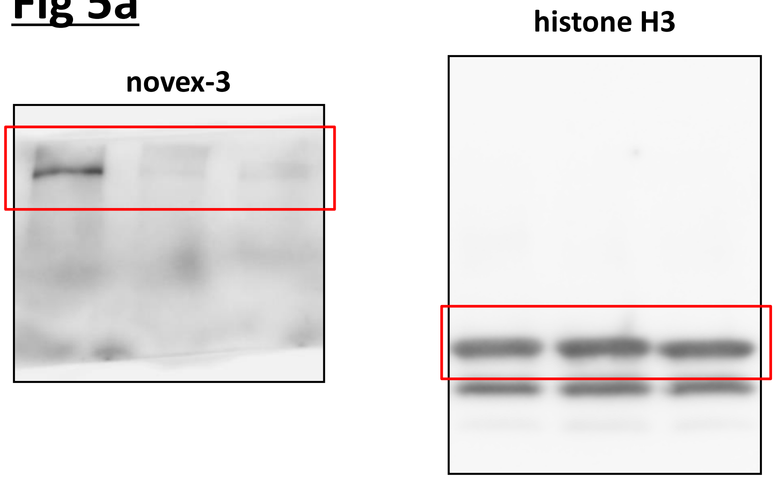


# Fig 5i

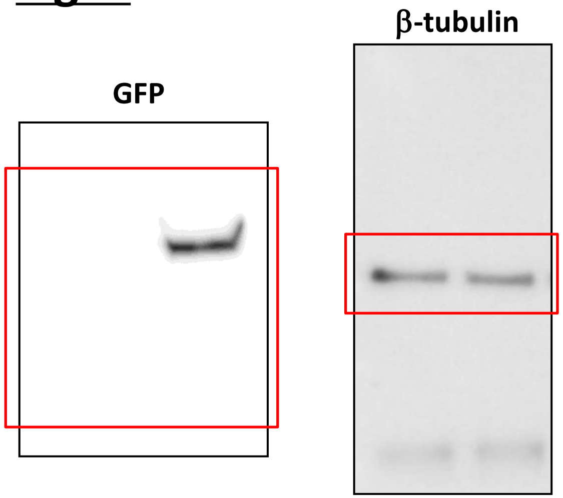

# Fig 8a

lamin A/C (pS392)

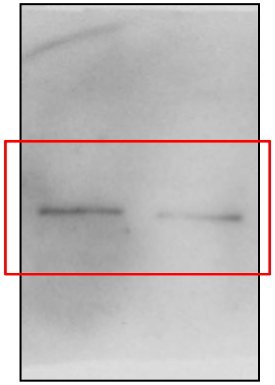

histone H3

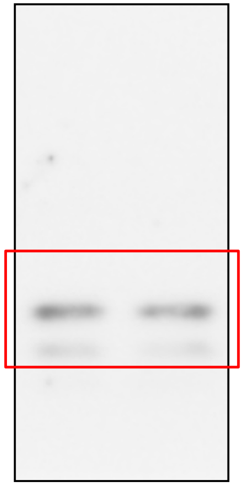

# Supplementary Fig S1

novex-3

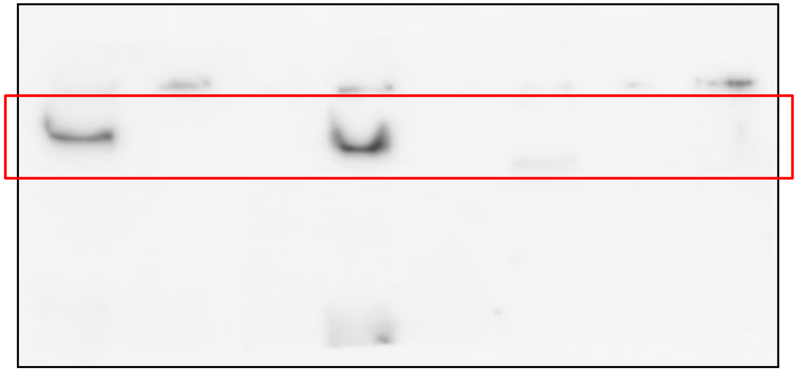

Supplement: Supplementary file 1 — Supplementary Information [file 41598_2018_30886_MOESM1_ESM.pdf]
